# Antineuronal antibody titres in autoimmune encephalitis: clinical implications for diagnosis and long-term immunotherapy

**DOI:** 10.3389/fimmu.2026.1771609

**Published:** 2026-03-10

**Authors:** Hanna Schwab, Josua Kegele, Markus C. Kowarik, Holger Lerche, Stephan Lauxmann

**Affiliations:** 1 Department of Neurology and Epileptology, Hertie Institute for Clinical Brain Research and Hertie Center of Neurology, University and University Hospital Tübingen, Tübingen, Germany; 2 Department of Neurology and Vascular Diseases, Hertie Institute for Clinical Brain Research and Hertie Center of Neurology, University and University Hospital Tübingen, Tübingen, Germany; 3 Neurocenter Klinikverbund Südwest, Böblingen, Germany

**Keywords:** antineuronal antibodies, autoimmune encephalitis, cerebrospinal fluid, immunotherapy, rituximab

## Abstract

**Introduction:**

The role of antineuronal antibody titres in the acute and long-term diagnostic and therapeutic management of autoimmune encephalitis (AE) remains unclear. In this retrospective monocentric cohort study, we aimed to (I) identify specific characteristics in antibody testing distinguishing AE from non-AE patients, (II) evaluate the prognostic significance of antineuronal antibody findings and (III) assess outcomes and long-term immunotherapy in patients with AE.

**Methods:**

Patients with suspected autoimmune-associated neuropsychiatric conditions underwent antineuronal antibody testing between 01/2017 and 03/2023. Patients with positive antibody tests were stratified into AE and non-AE groups based on the clinical criteria proposed by Graus and colleagues. Long-term outcomes, antibody titres, and therapeutic strategies were analysed in AE patients over a three-year follow-up period. Among 2,466 patients tested, 53 met the diagnostic criteria for AE.

**Results:**

In AE patients with paired serum and CSF samples (n = 44), antibodies were detectable in both serum and CSF in 55% of cases (n = 24), in serum only in 36% (n = 16), and in CSF only in 9% (n = 4). AE patients with poor outcomes (n=5) showed a trend toward higher median CSF titres in the acute phase and at four months post-onset compared to patients with good outcomes (n=14); however, differences were not statistically significant. Regarding long-term immunotherapy, rituximab-treated patients experienced fewer relapses than those receiving intravenous-immunoglobulins (IVIG; p-value = 0.02).

**Discussion:**

These exploratory results from a small, heterogeneous cohort require confirmation in larger, prospective studies. Based on our data regarding serum and CSF antibodies, in a resource- limited setting we propose a stepwise diagnostic approach starting with serum screening; in suspected anti-NMDAR-AE, initial paired serum/CSF testing remains essential. If antibodies are detected in serum, additional CSF antibody testing may provide diagnostic confirmation and help guide treatment decisions, as high acute-phase CSF titres may suggest poorer long-term outcomes; however, this potential prognostic value requires confirmation in larger, antibody-specific studies.

## 1 Introduction

Autoimmune encephalitis (AE) comprises a group of non-infectious, immune-mediated inflammatory diseases of the brain parenchyma with antibodies targeting neuronal cell surface, synaptic or intracellular antigens (1, 2). Patients with AE typically present with a subacute onset of working memory deficits, altered mental status, psychiatric symptoms or new-onset seizures. In 2016, diagnostic criteria were established by Graus et al. to facilitate rapid diagnosis in a clinical setting (3).

Antineuronal antibodies are considered rare in healthy individuals, however, data on their prevalence in the general population and among patients with neurological disorders remain limited (4, 5). In a study by Christian G. Bien, more than 10,000 samples from patients with suspected neuropsychiatric autoimmune conditions were tested for anti-neuronal antibodies, showing positive results in 5.3% of cases. Among patients with anti-NMDAR antibodies, 28% had detectable antibodies only in CSF, whereas LGI1 and CASPR2 antibodies were frequently found in serum only (33% and 48% respectively) (6). However, the possibility of clinical misdiagnosis increases when the CSF status remains unknown (7).

These findings raise questions about the significance of a positive antineuronal antibody test in patients with neurological disease. Furthermore, in clinical practice, the question remains whether initial serum testing could be sufficient (due to higher sensitivity for multiple antibodies) or whether serum/CSF pairs are mandatory in all cases.

In AE, antineuronal antibodies currently serve primarily a diagnostic role in confirming suspected AE cases (3). Due to limited availability of long-term data, one of the major challenges in managing patients with AE is determining both the indication for and the optimal duration of long-term immunotherapy, as its individual benefit remains difficult to predict (8, 9). Some evidence suggest that rituximab may reduce the risk of relapse (10). However, the role of antibody titres as biomarkers to guide long-term immunotherapy remains largely unclear (11), and as a result, titres are not routinely monitored in most patients with AE. A study on NMDAR-associated encephalitis demonstrated that patients with high antibody titres in serum and CSF had worse outcomes compared to those with low titres (12). Furthermore, the persistence of antibodies in CSF at 12 months was associated with poorer outcomes and higher relapse rates, although no significant differences were observed in long-term follow-up (13).

To better understand the significance of antineuronal antibody detection in patients with suspected autoimmune-associated neuropsychiatric conditions and confirmed AE, we collected data from patients who tested “positive” for antineuronal antibodies in our neurology laboratory. As our study focussed on antibody-positive individuals, it was not designed to comprehensively evaluate the sensitivity or specificity of antibody testing. The primary objectives were to compare antibody titres between AE and non-AE patients, to assess their association with long-term clinical outcomes in AE, and to explore the influence of different long-term immunotherapies on relapse risk. We explored whether higher CSF antibody titres might be associated with poorer long-term outcomes and whether rituximab could be more effective than IVIG in reducing relapse risk.

## 2 Materials and methods

### 2.1 Standard protocol approvals, registration and patients consents

Approval for this retrospective study was provided by the Ethics Committee of the Medical Faculty of Tuebingen with the project number 155/2023BO2. Prior to data collection, each patient was assigned a unique ID for pseudonymisation.

### 2.2 Study design and cohort

**Figure 1:**
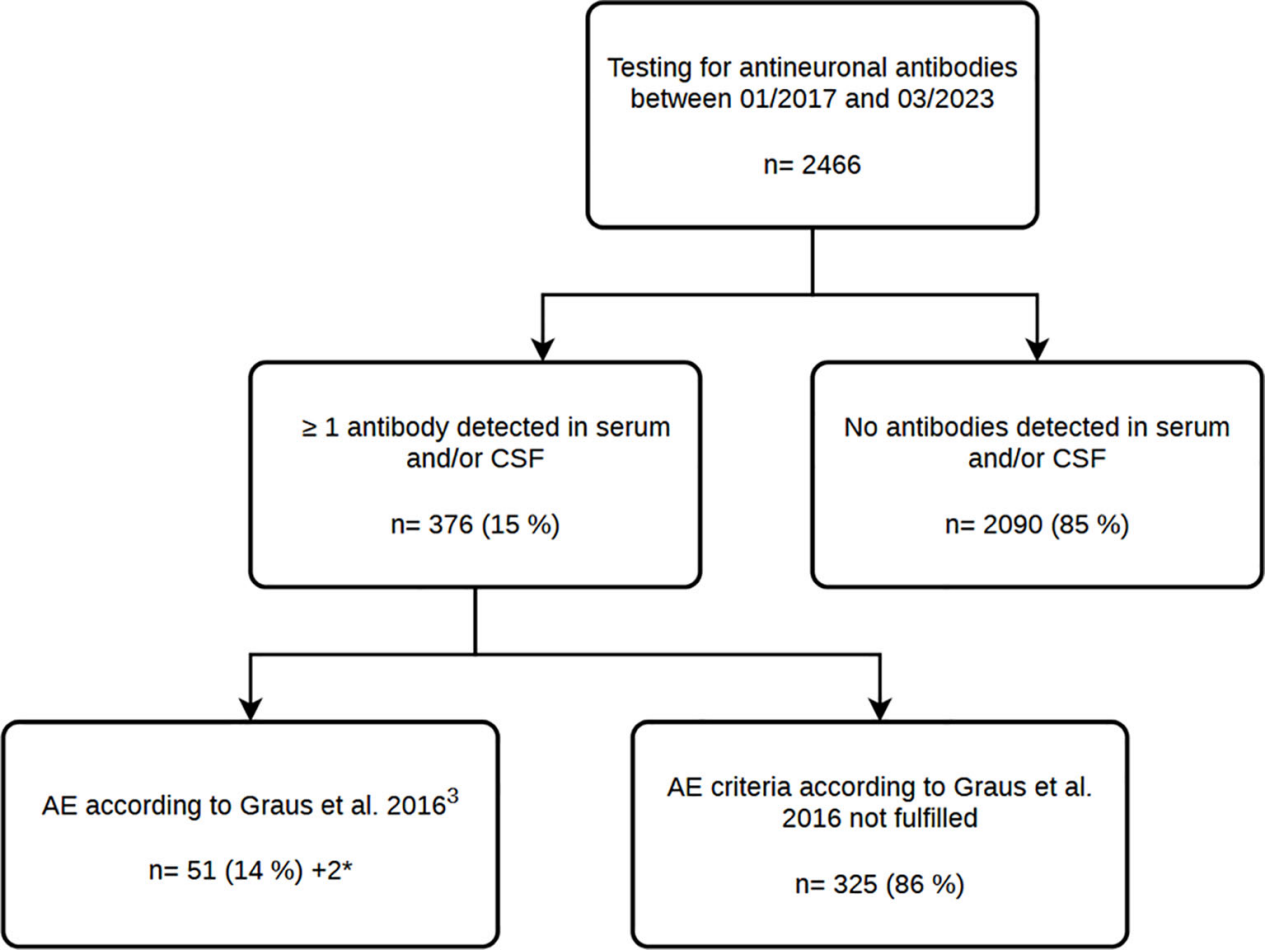
Flowchart of the study design and patient cohort. The number of patients is indicated by n. *Two patients who met the criteria for AE were identified through retesting at an external centre.

We analysed all patients who underwent antineuronal antibody testing in our laboratory between January 2017 and March 2023 with a suspected autoimmune-associated neuropsychiatric condition (**Figure 1**). Samples were obtained from serum or CSF and serum, following the test strategies shown in **Table 1**.

**Table 1:** Test strategies of antineuronal antibodies *IIFA testing only; **line-blot testing only.

Panel	Antibody-screening (panel 1)		Encephalitis mosaic (panel 2)
Method	IIFA (P1a)	Line-blot (P1b)	IIFA
Antibody	Hu	Hu	GlutamatR Type NMDA*
	Yo	Yo	GlutamatR Type AMPA1/2*
	Ri	Ri	CASPR2*
	Ma1, Ma2/Ta	PNMA2 (Ma-2/Ta)	LGI1*
	CV2	CV2	DPPX*
	Tr (DNER)	Tr (DNER)	GABAR*
	Amphiphysin	Amphiphysin	
	GAD-65	GAD-65	
	NMDAR*	Zic4**	
		Recoverin**	
		SOX1**	
		Titin**	

Line blot assays were performed using a commercial Euroimmun panel. IIFA was conducted on rat cerebellum and hippocampus tissue sections for intracellular antibodies, and on transfected cells for extracellular antibodies.

R, Receptor; P, Panel; IIFA, indirect immunofluorescence assay.

Intracellular antibodies were assessed using a commercial Line-Blot (Euroimmun) covering 12 antigens, and additionally by indirect immunofluorescence assay (IIFA) on rat cerebellum and hippocampus tissue sections. This approach was applied for Hu, Yo, Ri, Ma1, Ma2/Ta, CV2, Tr (DNER), Amphiphysin, and GAD65 antibodies. Additional intracellular antibodies, such as Zic4, Recoverin, SOX1 and Titin were assessed by Line-Blot only. Neuronal surface antibodies (NMDAR, AMPA1/2, DPPX, GABAR, LGI1, CASPR2) were tested by IIFA on transfected cells. No second confirmatory method was applied for these antibodies. Antibody titration was performed in accordance with the manufacturer’s instructions (Euroimmun). For cell-based assays, CSF samples were considered positive if a specific immunoreactivity was detectable in the undiluted sample, whereas serum samples were defined as positive at a screening dilution of 1:10. In Line-Blot analyses, borderline positive results were documented and included in the statistical evaluation. As patients were exclusively identified through positive antibody test results, cases of antibody-negative autoimmune encephalitis could not be captured, resulting in a selection bias.

Patients with a positive antibody test were stratified into AE and non-AE groups, according to the Graus criteria (3). If multiple samples from the same patient in the non-AE group were available during the study period, only the first antibody test result was included in the titre analysis. In patients with AE, the sample collected during the acute phase of the disease was used. Each patient with AE was assigned to a specific antibody; in cases of multiple antibodies, classification was based on the clinical phenotype. If very weakly positive antibodies were detected via Line-Blot and no clear clinical phenotype was identifiable, they were evaluated in favour of the stronger positive antibody.

**Figure 2:**
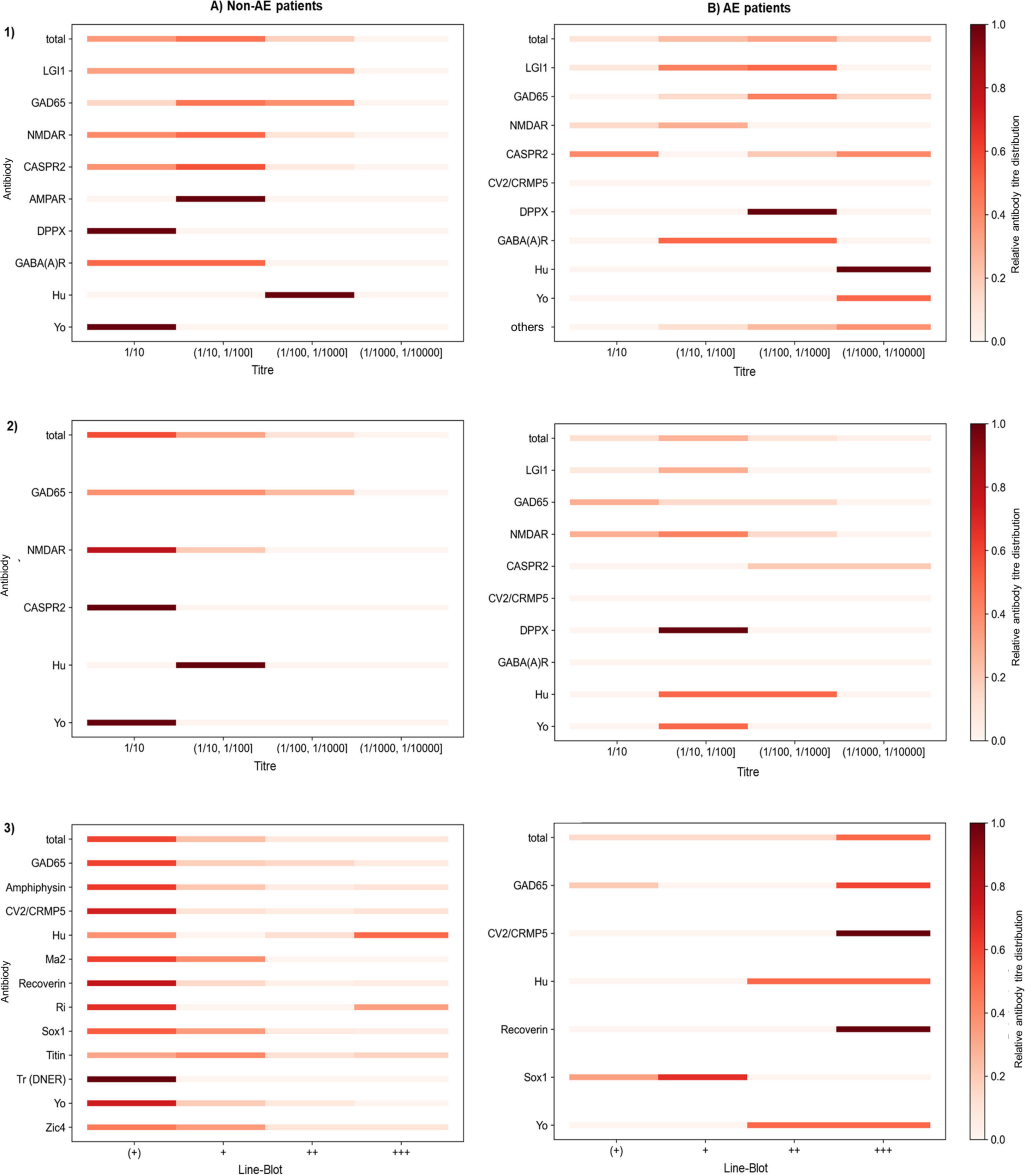
Colour progression chart illustrating the relative antineuronal antibody titres in **(A)** Non-AE patients and B) AE patients across three testing modalities: (1) serum (IIFA), (2) CSF (IIFA), and (3) serum (Line-Blot). Results are shown separately for **(A)** non-AE patients (n = 392; n (1) = 51, n (2) = 19, n (3) = 355) and **(B)** AE patients in the acute phase (n = 45; n (1) = 41, n (2) = 40, n (3) = 14). Each row represents a specific antibody, and each column a titre level. Colour intensity reflects the relative frequency of a given titre within each antibody target and test modality, with darker colours indicating more frequently observed titres. Non-AE patients predominantly exhibited low antibody titres, whereas AE patients more frequently showed higher titres in IIFA and Line-Blot.

**Figure 3:**
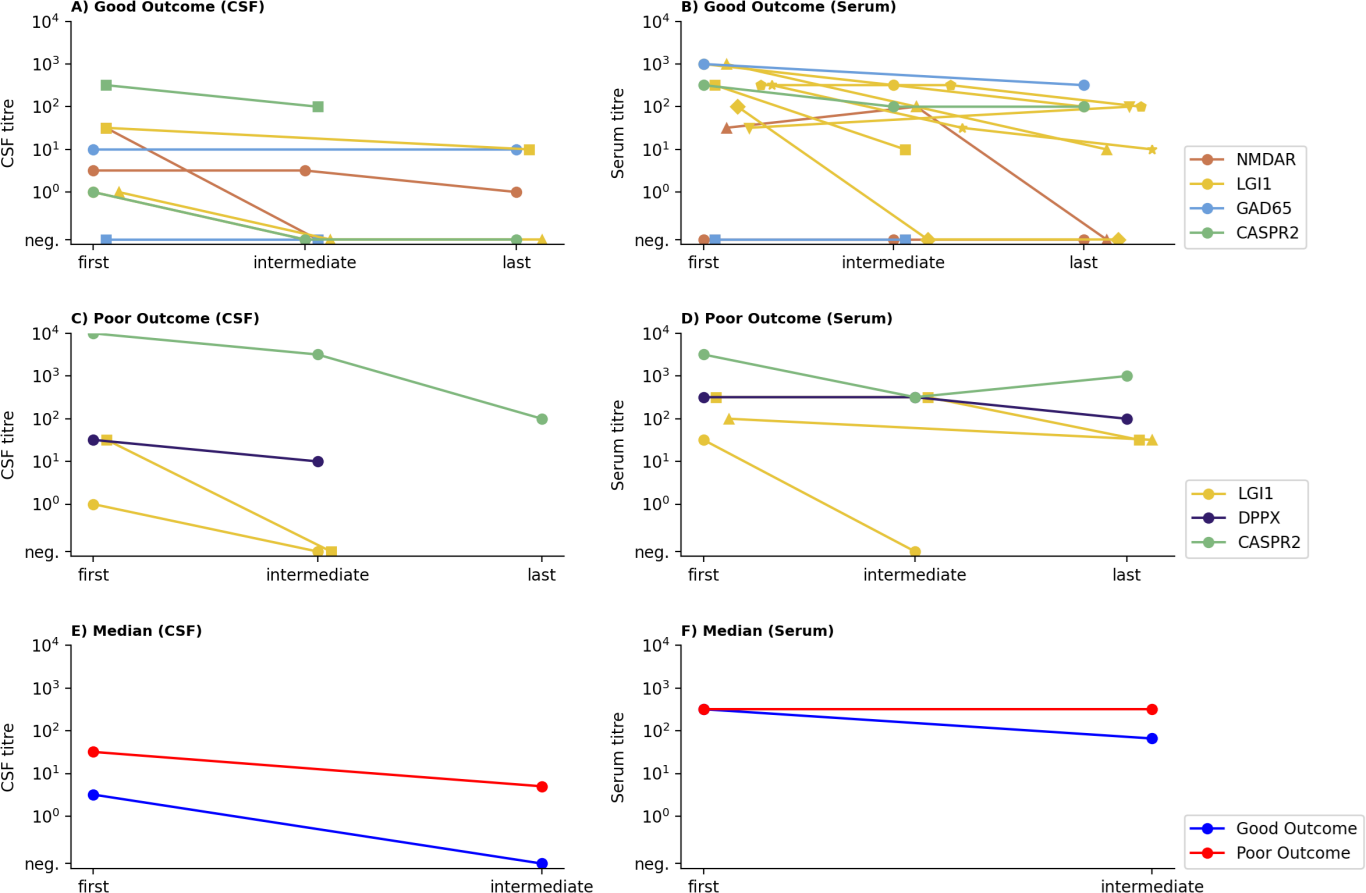
Time course of antibody titres according to patient outcome in CSF **(A, C)** and serum **(B, D)**. Antibody titres are shown on a logarithmic scale (y-axis) at defined time points (x-axis): first (acute phase), intermediate (2–6 months post onset), last (last available time point; median [IQR] in months: CSF 24.5 [11.3–35.5]; serum 28.0 [12.0–36.5]). Each patient is shown with a unique colour–symbol combination; colours indicate antibody targets. **(A–D)** Patients were stratified according to clinical outcome: **(A, B)** good outcome (CASE score 0–2, n = 14); **(C, D)** poor outcome (CASE score ≥3, n = 5). **(E, F)** Median antibody titres in patients with good (blue) versus poor (red) outcome in CSF **(E)** and serum **(F)**. Median CSF titres tended to be higher in patients with poor outcomes, whereas median serum titres did not differ markedly between outcome groups. Statistical comparisons between outcome groups at each time point were performed using the two-sided Mann–Whitney U test; no significant differences were observed for either CSF or serum titres (all p > 0.05). Titres were available from n = 9 (good, CSF), n = 14 (good, serum), n = 4 (poor, CSF), n = 5 (poor, serum) patients. neg.= negative.

To minimise potential sources of bias in the analysis, we applied exclusion criteria for acute-phase therapy, antibody titre, and outcome evaluations. Patients with preexisting epilepsy (n = 3), prior immunosuppressive therapy before the onset of autoimmune encephalitis (n = 2), and one patient with insufficient clinical data from the acute phase (n = 1) were included in the overall cohort description but excluded from acute-phase, antibody titre, and outcome analyses to reduce confounding related to preexisting seizure disorders or prior treatment effects. Furthermore, 13 patients were excluded from the long-term outcome analysis because no follow-up of at least 6 months (24 weeks) after initiation of immunotherapy was available, yielding a final cohort of 34 patients for outcome evaluation. For the analysis of antibody titres, 2 patients were excluded due to missing acute-phase titres, resulting in a total of 45 patients included for the acute titre analysis (**Figure 2**). For the assessment of titre evolution over time, an additional 18 patients were excluded due to missing follow-up measurements, yielding 27 patients included in the longitudinal analysis (**Figure 3**).

### 2.3 Data collection

Patient data were retrieved from our clinic database, which contains comprehensive records, including follow-up information, laboratory results, EEG and MRI findings. For the non-AE group - comprising all patients who did not fulfil the Graus criteria but tested positive for an antineuronal antibody - only demographic data and antibody titres were collected. For patients with AE, we collected detailed diagnostic, clinical and treatment related data (date of onset, preexisting conditions, antibody titres, CSF findings, MRI, EEG, seizure type and frequency, modified Rankin Scale (mRS), Clinical Assessment Score for AE (CASE score), and details of both acute and long-term immunotherapy. If specific clinical, diagnostic, or treatment information was not explicitly documented in the medical record, it was interpreted as absent. Data were collected during the acute phase and at each follow-up appointment, which was defined as any instance when a patient with AE returned for consultation or was hospitalised.

### 2.4 Operational definitions and outcome measures

The term “autoimmune-associated neuropsychiatric condition” was defined as any patient in whom antineuronal antibody testing was clinically indicated, i.e., those presenting with neuropsychiatric symptoms suggestive of an autoimmune aetiology. This cohort included all patients tested for antibodies within the defined study period. The classification of AE patients was performed according to the Graus criteria for “possible AE” (3). Patients who fulfilled these criteria and additionally tested positive for an antibody in serum, CSF, or both were included in the AE cohort. To evaluate the clinical course of patients with AE, the modified Rankin Scale (mRS) and the Clinical Assessment Score for AE (CASE score) were assessed both during the acute phase and at each follow-up visit. The CASE score consists of nine items: seizure, memory impairment, psychiatric symptoms, consciousness, speech problems, dyskinesia/dystonia, gait instability, ataxia and brainstem dysfunction. Each item is rated on a scale from 0 (no symptoms) to 3 (severe symptoms), resulting in a total score ranging from 0 to 27, with higher scores indicating greater disease severity and symptoms burden (14, 15).

Short-term outcome (after the acute phase) was defined as the CASE score recorded at least four weeks after the initiation of immunotherapy, but before the six-month mark. Long-term outcome was based on the most recent CASE score obtained at six months or later following therapy initiation. Outcome analysis included only patients with at least one follow-up visit within the defined timeframe.

Patients were classified into three long-term outcome categories based on clinical functional impairment: (1) good outcome (CASE score 0-2); (2) Poor outcome (CASE score ≥3); (3) Relapse (an increase of ≥3 points in the CASE score compared to the previous follow-up). One patient was retrospectively identified as having relapsed despite missing the follow-up visit and was included in the relapse group.

For the start of acute treatment, early immunotherapy was defined as initiation of treatment within four weeks of symptom onset, whereas late immunotherapy referred to treatment initiated more than four weeks after symptom onset. Long-term immunotherapy was defined as any immunomodulatory treatment intended to provide a sustained immunosuppressive effect, administered after the corticosteroid pulse (if applicable), regardless of whether it was started during the acute phase or shortly thereafter. All long-term immunotherapies were continued beyond the acute phase.

### 2.5 Statistical analysis

Data were collected and managed using Microsoft Excel (Microsoft Corporation, Redmond, WA, USA), initially for the entire cohort, followed by subsets for all positive patients and those with AE. Qualitative data were summarised as frequencies and presented as absolute and relative values (n, %), while quantitative data were described using median and interquartile ranges. Further statistical analyses were performed using the Python programming language within the Visual Studio Code development environment. These analyses include colour-coded plots and line graphs for titre evaluation, including Mann-Whitney U tests to compare titres between different outcome groups and a Kaplan-Meier curve to compare relapse risk across different long-term immunotherapies using the log-rank test. Patients lost to follow-up were censored at the time of their last documented clinical assessment and included in the Kaplan-Meier analysis accordingly. For the relapse analysis across long-term immunotherapy groups, patients were assigned to either the Rituximab, IVIG, or no therapy group based on the treatment they received prior to a potential relapse. Patients were included in the Rituximab or IVIG group even if they had received additional immunomodulatory agents. Patients who received both Rituximab and IVIG, or other forms of long-term immunotherapy not including either agent, were excluded from the Kaplan-Meier analysis. For antibody titres, we investigated differences between patients with good versus poor outcomes at three time points: First (first measured titre), Intermediate (Representative titre during months 2–6), Last (last measured titre). Group comparisons were performed using the Mann–Whitney U test, a non-parametric, two-sided test chosen due to small sample sizes and non-normal data distribution. Negative samples were scored as 1, so that their values remain small when transformed logarithmically.

### 2.6 Data availability

The authors confirm that all data from this study can either be found in the appendix or be obtained upon reasonable request.

## 3 Results

### 3.1 Study cohort

Between January 2017 and March 2023, a total of 2466 patients with suspected autoimmune-associated neuropsychiatric condition were tested for antineuronal antibodies. Among these, 376 patients had at least one antibody detected in serum and/or CSF, with 51% being male and 49% female. However, gender distribution varied by antibody type; for example, antibodies against NMDAR were more common in female patients, while antibodies against SOX1 were more prevalent in male patients (**Supplementary Figure 2**). In the overall cohort, antibodies against GAD65, Recoverin, SOX1, Yo and Titin were the most frequently detected. The median age at testing was 62 years. Patients with antibodies against NMDAR were the youngest, with a median age of 42 years. Across the entire cohort, a total of 453 antibodies were detected, with 62 patients presenting with multiple antibodies. The most common antibody combinations included GAD65 (n=23), Recoverin (n=22), SOX1 (n=15), Titin (n=12) and Amphiphysin (n=10) (**Supplementary Figure 3**).

When an antineuronal antibody was detected in serum, CSF or both, AE occurred in 13.6% (n=51) of cases according to Graus criteria. AE was particularly common in patients with LGI1 (82.4%) and NMDAR antibodies (38.9%).

### 3.2 Clinical characteristics of patients with AE in the acute phase

Among patients with AE, the most frequently detected antibodies were LGI1 (n=15; 28%), GAD65 (n= 10; 19%), NMDAR (n=7; 13%) and CASPR2 (n=5; 9%) (**Supplementary Figure 4**). The cohort consisted of 49% (n=26) male and 51% (n=27) female patients. All AE patients with antibodies against NMDAR were female, whereas all patients with antibodies against CASPR2 were male. The median age at symptom onset was 59 years, with anti-NMDAR patients being the youngest (median age 31 years) and anti-CASPR2 patients being the oldest (median age 66 years) (**Table 2**).

**Table 2:** Clinicodemographic characteristics and diagnostic findings of AE patients: total cohort and subgroups with LGI1, GAD-65, and NMDAR antibodies.

	Total	LGI1	GAD65	NMDAR
Overall cohort: Patients, n (%)	53 (100)	15 (28)	10 (19)	7 (13)
Age at diagnosis (y), median (IQR)	59 (45-71)	62 (53-72)	62 (34-72)	31 (20-35)
Female sex, n (%)	27 (51)	7 (47)	6 (60)	7 (100)
Time to therapy (we), median (IQR)	5,4 (2-23)	7 (5-20)	5 (2-23)	1 (1-3)
Time hospitalised (d), median (IQR)	9 (6-18)	8 (5-12)	7 (5-15)	26 (14-30)
Long-term outcome available, n (%)	34 (64)	13 (87)	6 (60)	6 (86)
Relapse, n (%)	10 (29)	1 (8)	4 (64)	3 (50)
Clinical and diagnostic findings in the acute phase of AE†				
Acute phase evaluation: Patients, n (%)	47	14	8	7
MRI performed, n (%)	46 (98)	13 (93)	8 (100)	7 (100)
MRI not suggestive for AE, n (%)	24 (52)	2 (15)	7 (88)	5 (71)
EEG performed, n (%)	40 (85)	14 (100)	7 (88)	6 (86)
Normal EEG, n (%)	14 (35)	3 (21)	2 (29)	2 (33)
Diffuse slowdown, n (%)	6 (15)	2 (14)	1 (14)	2 (33)
Focal temporal slowing, n (%)	5 (13)	1 (7)	1 (14)	2 (33)
Epileptiform discharges, n (%)	16 (40)	6 (43)	5 (71)	2 (33)
Extreme delta brush, n (%)	1 (3)	–	–	1 (17)
CSF analysis performed, n (%)	44 (94)	14 (100)	7 (88)	7 (100)
Cell count (x/µl), median (IQR)	3 (1-9)	2 (1-4)	4 (2-6)	19 (9-43)
Protein (mg/dl), median (IQR)	43 (33-57)	42 (35-57)	43 (32-56)	35 (22-62)
BBB dysfunction present, n (%)	14 (32)	5 (36)	2 (29)	2 (29)
Q Albumin (1x10^-3^), median (IQR)	6 (5-9)	8 (5-9)	5 (5-7)	5 (4-9)
Q IgG (1x10^-3^), median (IQR)	3 (2-4)	3 (2-5)	2 (2-4)	3 (2-5)
mRS score, median (IQR)	3 (2-3)	3 (2-3)	3 (2-3)	3 (3-4)
CASE score, median (IQR)	5 (4-7)	5 (3-7)	4 (4-6)	7 (5-11)
Seizures, n (%)	28 (60)	11 (79)	5 (63)	3 (43)
Focal aware, n (%)	15 (54)	9 (82)	1 (20)	–
Focal impaired awareness, n (%)	14 (50)	5 (45)	2 (40)	1 (33)
Focal to bilateral tonic clonic, n (%)	5 (18)	1 (9)	–	1 (33)
Focal non-motor, n (%)	8 (29)	4 (36)	–	–
Focal motor, n (%)	10 (36)	4 (36)	1 (20)	–
Generalised tonic-clonic, n (%)	2 (7)	–	–	2 (67)
Unknown onset motor, n (%)	1 (4)	–	1 (20)	–
Facio-brachial dystonic seizure, n (%)	5 (18)	5 (45)	–	–
Seizures (per mo), median (IQR)	4 (1-83)	105 (90-600)	1 (1-7)	1 (1-2)
Memory impairment, n (%)	36 (77)	12 (86)	6 (75)	5 (71)
Psychiatric symptoms, n (%)	17 (36)	6 (43)	2 (25)	5 (71)
Impairment of consciousness, n (%)	11 (23)	1 (7)	1 (13)	4 (57)
Speech disorder, n (%)	27 (57)	6 (43)	6 (75)	5 (71)
Dyskinesia/dystonia, n (%)	9 (19)	1 (7)	2 (25)	2 (29)
Gait instability and ataxia, n (%)	20 (43)	5 (36)	3 (38)	3 (43)
Brainstem dysfunction, n (%)	3 (6)	–	1 (13)	1 (14)
Paresis, n (%)	1 (2)	–	–	–
Autonomic dysfunction, n (%)	4 (9)	4 (29)	–	–

† Percentages refer to the number of patients included in the acute-phase evaluation. Six Patients were excluded from subgroup analysis as described in the Methods section.

y, years; mo, months; we, weeks; d, days; IQR, interquartile range; BBB, Blood Brain Barrier; Q, Quotient; CASE, Clinical Assessment Score for AE; n, number of patients.

The average hospital stay for patients with AE was nine days, with anti-NMDAR patients having the longest median hospitalisation (26 days). Intensive care unit (ICU) admission was required in only 11% of cases. Prior to AE onset, all patients showed a mRS between 0 and 1, indicating good baseline health. During the acute phase, the mean mRS was 3 and the median CASE score was 5. Most patients presented with memory loss (77%), epileptic seizures (60%), speech disorders (57%) and gait instability or ataxia (43%). Psychiatric symptoms were present in 36% and impaired consciousness in 23% of patients. The most common seizure types, according to ILAE classification (16) were focal aware (32%) and focal unaware (30%) seizures. Faciobrachial dystonic seizures were exclusively observed in five of 15 patients with LGI1 antibodies.

A correlation was found between CASE and mRS score in both the acute phase and the last follow-up (r= 0.73; r=0.83; p-value <0.01) (**Supplementary Figure 5**). Therefore, the CASE score was used for follow-up assessments.

### 3.3 Antineuronal antibodies in AE and non-AE patients

**Figure 4:**
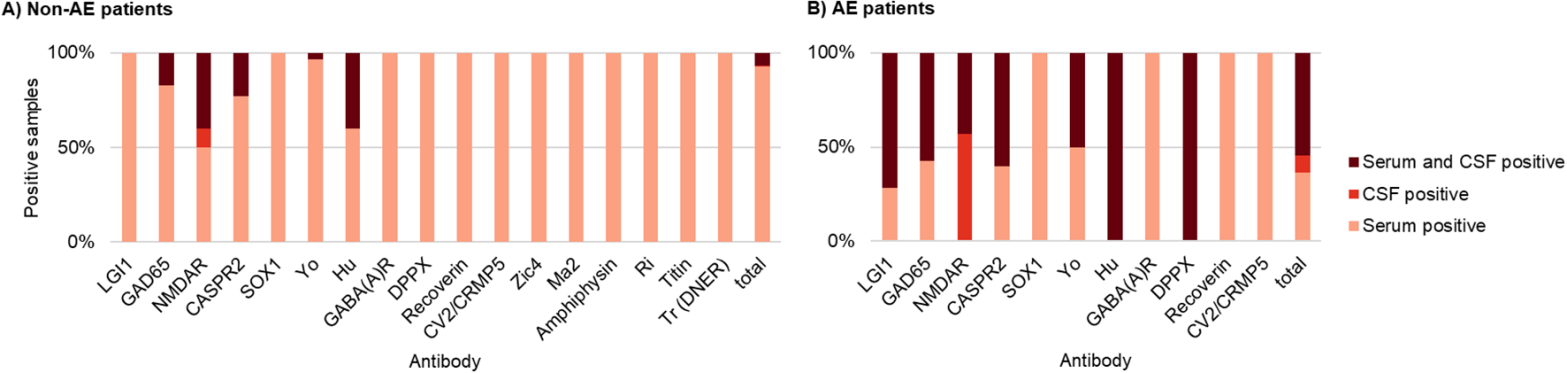
Antibody positivity in paired serum/CSF samples from **(A)** non-AE patients (n=262) and **(B)** patients with AE (n=44). For each patient, serum and CSF were tested, and antibody positivity was classified as serum only, CSF only, or in both compartments. For each antibody target, stacked bars summarise all antibody-positive patients and display the proportion of patients with serum-only, CSF-only, or combined serum/CSF positivity, with each bar normalised to 100%. Antibody positivity was defined by detection using IIFA, line blot, or both, according to the test strategies outlined in **Table 1**. Among AE patients with paired samples, 55% (n = 24) showed antibody positivity in both serum and CSF, 36% (n = 16) showed isolated serum positivity - most frequently involving LGI1 (n = 4), GAD65 (n = 3), and SOX1 (n = 3) - and 9% (n = 4) showed isolated CSF positivity (exclusively NMDAR antibodies). Non-AE patients with paired serum/CSF samples exhibited isolated serum positivity in more than 90% of cases.

In patients with AE and serum/CSF pairs tested, 55% (n=24) were positive in both the serum and the CSF. Isolated serum positivity was found in 36% (n=16) of patients, primarily involving antibodies directed against LGI1 (n= 4), GAD65 (n= 3) and SOX1 (n= 3). Isolated CSF positivity was observed in 9% of cases (n= 4), exclusively for NMDAR antibodies. In contrast, non-AE patients with paired serum/CSF samples showed isolated positive serum samples in more than 90% of the cases (**Figure 4**).

In patients with AE, IIFA titre values ranged from 0 to 1/10,000 in both serum and CSF. Overall, serum titres occurred predominantly in the medium- to high-titre range between 1/10 and 1/1,000 whereas CSF titres were generally lower (1/10 to 1/100) (**Figure 2**). Line-Blot testing was negative in only one anti-GAD65 AE patient; notably, this patient showed high IIFA titres (serum 1/1000; CSF 1/100). All remaining patients demonstrated positive Line-Blot findings, predominantly with strongly positive results. In contrast, non-AE patients generally showed lower titres in the IIFA and the blot showed only marginal positive results in the majority of samples. For example, of more than 40 positive Yo-antibodies in Line-Blot of non-AE patients, only two were confirmed via IIFA (serum). An exception was SOX1 antibodies, which showed weak positivity even in patients which fulfilled the diagnostic criteria for AE.

### 3.4 Therapy and outcome

Out of 47 AE patients, 42 (89%) received immunomodulatory therapy in the acute phase. Most patients received steroid pulse therapy with 1000–2000 mg of intravenous methylprednisolone per day for three to five days. Only two patients received IVIG monotherapy instead. Combination therapy was administered to 13 patients, most commonly including additional immunoglobulins. Five patients (11%) received no therapy in the acute phase. (**Supplementary Table 10**) The average time from symptom onset to initiation of immunotherapy was 5.4 weeks. In long-term follow-up, patients who received early immunotherapy had a lower median CASE score (CASE score = 1) compared to those who received late immunotherapy (CASE score = 2), although the median mRS was 1 in both groups (**Supplementary Figure 6**). Patients with antibodies against extracellular antigens showed better median CASE scores throughout the disease course than those with antibodies against intracellular antigen targets (**Supplementary Figure 7**).

Long-term outcome was available for 34 patients. After a median follow-up of 2.9 years (range 1.3-4.0 years), 50% (n=17) of the patients with AE showed a good outcome, 21% (n=7) had a poor outcome and 29% (n=10) experienced relapse. For long-term immunotherapy, a total of 14 different regimens were used throughout the entire follow-up period (including before and after any potential relapse) in 27 out of 34 patients, while 21% (n=7) did not receive long-term immunotherapy at any time (**Supplementary Table 11**). Over the course of the whole observation period, 47% (n=16) received rituximab (as monotherapy or in combination), and 32% (n=11) received alternative immunosuppressive therapies.

**Figure 5:**
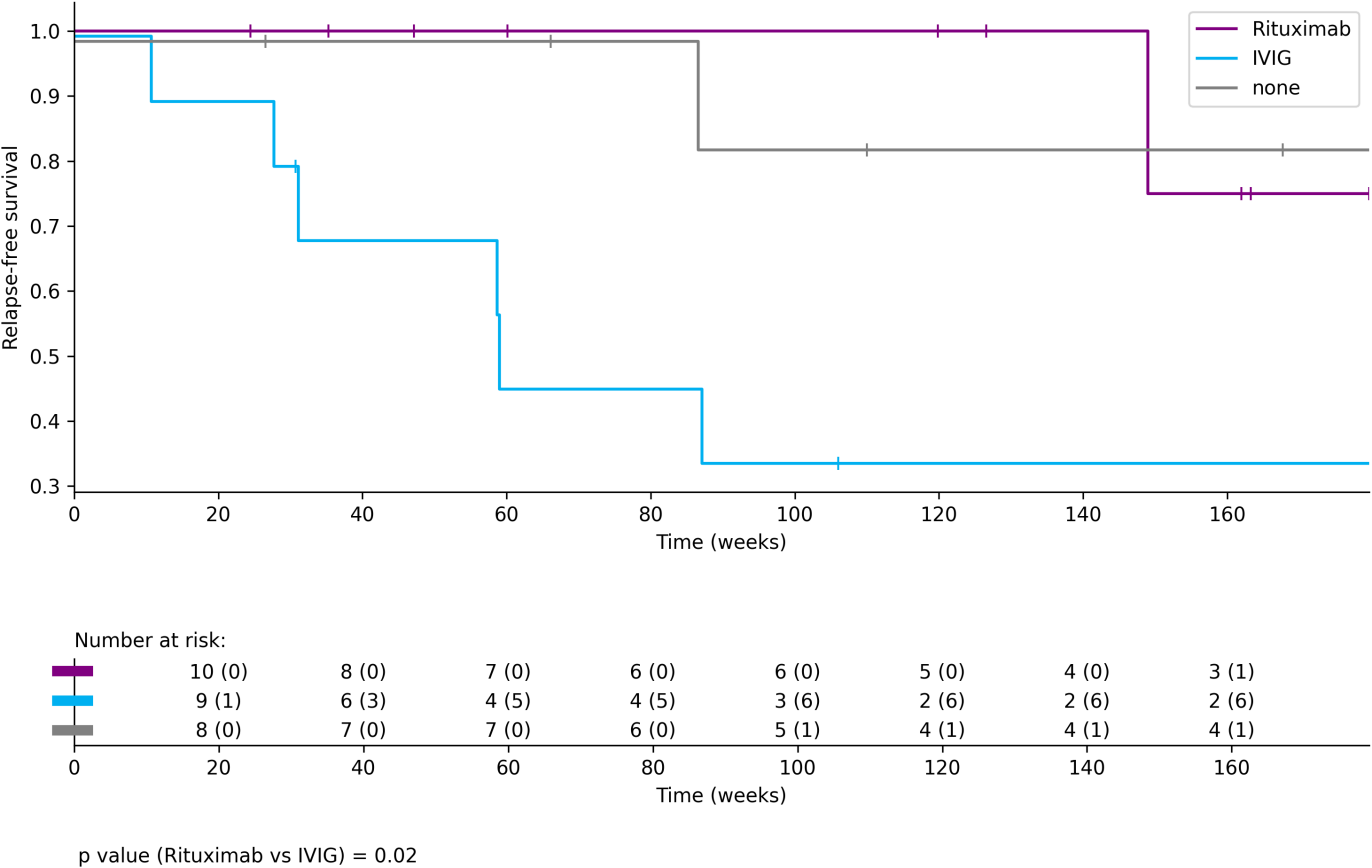
Kaplan-Meier analysis of relapse-free survival in patients receiving Rituximab treatment (purple, n=10), IVIG (blue, n=10) or no long-term immunotherapy (grey, n=8), with a total of 27 patients at baseline. Patients treated with Rituximab (purple) showed significantly fewer relapses compared to those receiving IVIG (blue) (p=0.02). In the IVIG group, patients received a median of 8.5 IVIG cycles (IQR 3.5 – 12.75). Relapse-free survival in the no-treatment group was comparable to that observed in Rituximab-treated patients.

A Kaplan-Meier analysis comparing selected long-term immunotherapies (Rituximab, IVIG, or no therapy) demonstrated a lower relapse rate in patients treated with rituximab (p-value = 0.02; **Figure 5**). The analysis included only patients who received Rituximab, IVIG, or no long-term immunotherapy during the disease course and prior to any relapse. Patients who received both rituximab and IVIG, or any other form of long-term immunotherapy, were excluded (n=6), resulting in a final sample of n=27 for this analysis. Most patients included in the Kaplan-Meier analysis had antibodies against extracellular targets, with the exception of two patients in the no- long-term immunotherapy -therapy group who were tested positive for Ma2 and CV2/CRMP5 antibodies and four patients with anti-GAD65 antibodies in the group treated with IVIG. Among patients treated with Rituximab, only one out of ten (10%) experienced a relapse, which occurred more than 140 weeks after initiation of therapy. In contrast, six out of nine patients (67%) receiving IVIG relapsed, including three patients with GAD-65 antibodies. In the group without long-term immunotherapy, two of eight patients (25%) experienced a relapse (both with antibodies targeting extracellular antigens), one of whom relapsed 213 weeks after the initiation of immunotherapy (not shown in **Figure 5**). The median CASE score in the acute phase of AE was 3.5 (range 2.75 - 6.0) in patients who did not receive long-term immunotherapy; compared to 7.0 (range 5.0 - 8.75) in the rituximab group and 5.5 (range 5.0 - 6.75) in the IVIG group.

Titres at multiple time points were available for 27 AE patients, allowing assessment of their evolution over the disease course. At final follow-up, 14 patients had a good outcome, 5 a poor outcome, and 8 experienced relapse. No clear correlation between acute phase antibody titres and disease severity scores (CASE and mRS) at the time of sampling was observed, with weak and inconsistent correlation coefficients for CASE (CSF −0.015, serum −0.193) and mRS (CSF 0.222, serum −0.44). **Figure 3** illustrates the course of antibody titres in patients with monophasic disease (good or bad outcome, n = 19), while **Supplementary Figure 8** shows the corresponding data for patients with relapsing disease (n = 8). The first sample was obtained during the acute phase of AE, the intermediate sample at least 2 months after disease onset, and the final sample at last follow-up. In the early phase of the disease, AE with poor outcomes (n=5) showed a higher median CSF titre than those with good outcomes (n=14). Median CSF titres remained elevated at the intermediate time point in the poor outcome group, whereas they decreased in the good outcome group. In serum, median titres were comparable between outcome groups at disease onset, with a slight decrease observed over time in the good outcome group; titres remained in the positive range for both groups. Statistical comparisons were performed using the two-sided Mann-Whitney U test, which revealed no significant differences between outcome groups at any of the three assessed time points (first, intermediate, last in serum and CSF; all p >0.05). Among relapsing patients (n=8), CSF titres increased at relapse in some but not all cases (**Supplementary Figure 8**). Patients with regular antibody monitoring (n=4) showed persistent titres prior to their relapse. Notably, an increase in serum titres at relapse was observed in only one case.

## 4 Discussion

In this real-world data study, we set out to (I) identify specific characteristics in antibody testing distinguishing AE from non-AE patients in a large cohort of patients with suspected autoimmune associated neuropsychiatric conditions, (II) evaluate the prognostic significance of antineuronal antibody findings, and (III) assess long-term outcomes and implications for long-term immunotherapy in patients with AE.

Our data are roughly in line with the previously published distribution of positive antineuronal antibodies in patients with suspected autoimmune-associated neuropsychiatric conditions, although much lower antibody positivity rates (approximately 5%) have been reported compared to the 15% positivity rate observed in our cohort (6, 17). This discrepancy may be explained by the exclusion of low-titre antibodies in prior studies, as well as differences in pre-test probability due to centre-specific selection criteria for antibody testing. Similarly, lower rates of multiple antibody positivity have been reported before, which could be attributed to our broader antibody panel that included Recoverin, SOX1 and Zic4 antibodies. Especially the before mentioned antibodies were frequently detected in our patient population with multiple antibodies (17).

The sensitivity of antibody detection in serum and CSF varies substantially depending on the antibody. Reduced sensitivity in CSF for antibodies against LGI1 and CASPR2 has been previously reported (6, 18)⁠. Consistent with these findings, approximately one third of our AE patients with antibody detection only in serum predominantly involve antibodies against LGI1, GAD65, SOX1, and CASPR2. Exceptions are AE patients with positive NMDAR antibodies which have repeatedly shown increased sensitivity in CSF (12, 19). Consistent with these studies, NMDAR antibodies were detected exclusively in CSF multiple times in our AE cohort.

Based on our data, we propose a clinical approach that specifically addresses antibody testing, taking into account the increased sensitivity of many serum antibodies (20) to identify patients who may benefit from additional CSF antibody screening in a resource – limited setting. Importantly, lumbar puncture with comprehensive CSF analysis (including routine parameters such as cell count and infectious work-up) remains necessary in patients with suspected encephalitis to exclude relevant differential diagnoses, irrespective of whether CSF antibody testing is performed. If autoimmune encephalitis is a relevant differential diagnosis in a patient, initial antibody screening could be performed as a first “screening” step with serum antibody testing alone.

In two scenarios, this “screening” could then be supplemented by CSF testing:

1. If the criteria for “probable anti-NMDA receptor encephalitis” are met, as defined by Graus et al., CSF antibody testing should be performed immediately (3).
2. In patients with positive serum antibodies, CSF testing may be used to confirm the diagnosis and guide therapeutic decisions throughout the disease course.

Following this proposed algorithm, patients with negative serum antibody results—particularly in the absence of clinical features suggestive of anti-NMDAR encephalitis—and in whom the clinical suspicion of autoimmune encephalitis decreases during the diagnostic work-up (e.g., due to emerging evidence for an infectious aetiology) would therefore not require additional CSF antibody testing. If, however, doubts persist after the initial evaluation, CSF testing should still be performed to avoid missing cases of anti-NMDAR encephalitis in which the diagnostic criteria were not initially fully met. Graus et al. have emphasised the potential for false-positive results in serum, and in the case of anti-NMDAR antibodies, false-negative results, especially at low titres (21). The challenge of false-negative serum findings in NMDAR encephalitis is addressed by paired serum/CSF testing in patients meeting the criteria for probable NMDAR-AE (scenario 1) while subsequent CSF testing in seropositive patients (scenario 2) provides additional specificity, diagnostic confirmation and potential therapeutic guidance. Comprehensive NMDAR antibody screening should especially be considered in young women with compatible clinical features or in the presence of a teratoma, given the high prevalence of NMDAR-antibodies in this population (22). Given the substantial costs of antibody testing in routine clinical practice, this stepwise approach may allow broader access to clinically relevant antibody screening while maintaining sensitivity: initial serum testing identifies patients at lower cost, followed by confirmatory CSF testing (if serum antibodies are positive) to increase specificity. This approach is intended as a pragmatic clinical framework to identify patients earlier, reduce the risk of missed diagnoses, and allow targeted use of CSF antibody testing in those most likely to benefit. In routine clinical practice, acute immunotherapy with high-dose corticosteroids is generally initiated promptly in patients with a strong clinical suspicion of autoimmune encephalitis, without awaiting antibody test results, indicating that the stepwise testing approach we propose is unlikely to delay the initiation of acute therapy. In settings without economic constraints, simultaneous serum and CSF testing in all suspected cases would remain the preferred strategy.

As expected, anti-GAD65-antibodies were found predominantly in the medium-titre range in the non-AE group, consistent with their clinical relevance typically arising only when levels are increased by a factor of 100 to 1000 (3, 18). It should be noted that, for GAD65-antibodies, quantitative assays such as radioimmunoassay (RIA) with established serum and CSF cut-offs are recommended to distinguish low-titre, often clinically irrelevant reactivity from high-titre, potentially pathogenic antibodies. In the present study, GAD65 antibodies were assessed using IIFA and Line-Blot (**Table 1**) as part of routine diagnostics, which allows only semi-quantitative interpretation and represents an important methodological limitation when comparing the GAD65 titres in the AE and non-AE group (23).

Previous studies have reported a high rate of false-positive results in Line-Blot testing, particularly for antibodies such as Zic4 and Yo (11, 24–26). In our cohort, we observed a substantial number of very weakly positive antibody results in Line-Blot, especially among non-AE patients. For instance, only two out of more than 40 Yo-antibody-positive Line-Blot results among non-AE patients could be confirmed by IIFA, with both tests conducted in serum. Notably, more than half of the AE patients with paired serum/CSF samples demonstrated antibody positivity in both compartments, whereas over 90% of non-AE patients showed isolated serum positivity (**Figure 4**). This suggests a high rate of false-positive findings in serum-only samples, especially at low titres, underscoring the importance of confirmatory CSF testing (7). AE patients typically exhibited strong positive results in Line-Blot testing and higher antibody titres in both serum and CSF assessed by IIFA (**Figure 2**). Given the potential pathogenic role of antineuronal antibodies, strongly positive Line-Blot results and high IIFA titres should be carefully evaluated in the context of a possible AE diagnosis.

An exception to this trend is SOX1 antibodies. Prior studies have reported that SOX1 antibodies are not reliably detected by Line-Blot, even when clearly positive in IIFA. In such studies, very weakly positive Line-Blot results were often classified as negative in order to reduce the risk of false-positive findings (24). However, this approach may increase the false-negative rate and lead to underdiagnosis of SOX1 antibody-associated disease if only tested in Line-Blot, as SOX1 antibodies were frequently weakly positive in our patients with confirmed AE (**Figure 2**). These findings highlight the need to assess the sensitivity and specificity of IIFA and Line-Blot individually for each antibody. The combined use of both techniques, as is already common practice, remains valuable.

High antibody titres at the disease onset have previously been associated with poorer long-term outcomes in patients with AE (12, 13). Consistent with these reports, patients in our cohort with poor outcomes exhibited a higher median CSF antibody titre at diagnosis. Moreover, in these patients, the median antibody titre remained detectable for three to six months after initiation of treatment. In contrast, patients with favourable outcomes showed no detectable median antibody titre in CSF at follow-up. However, we found no clear correlation between acute phase antibody titres and the level of the CASE or mRS scores. The statistical analysis using Mann–Whitney U tests at three predefined time points (first/acute, intermediate, and last follow-up) revealed no significant differences in CSF or serum titres between outcome groups (all p > 0.05). Accordingly, these findings do not support a statistically significant association between antibody titres and clinical outcome and should be interpreted as descriptive observations only. Routine serial antibody titre monitoring is not standard clinical practice, and therefore the small sample size in our study limits the generalisability of these results. Additionally, serum antibody titres are particularly difficult to interpret, as treatments such as plasmapheresis or IVIG can reduce serum antibody levels without substantially affecting CSF titres. As previously described, serum antibody titres at disease onset did not show prognostic relevance in our cohort either (27). Overall, the relationship between CSF antibody titres and clinical outcomes remains debated. While some studies have found a significant correlation (12, 28), others did not demonstrate such an association (29). It has also been suggested that the brain may act as an ‘immunoprecipitator’ of neuronal surface antibodies, which raises questions about how accurately CSF titres reflect the actual burden of pathogenic antibodies within the central nervous system (30). Additionally, persistent antibody positivity has been reported in some patients who have clinically recovered, underscoring the need for cautious interpretation of antibody persistence in relation to clinical outcome (12). Taken together, the prognostic value of antibody titres remains uncertain, and further investigation in larger, antibody-specific cohorts is needed to assess whether CSF titres could serve as clinically useful biomarkers.

The importance of early diagnosis and subsequent immunotherapy in AE is well known (2, 3) and our patient cohort also showed moderately improved outcomes with early treatment initiation. However, treatment regimens for both acute and long-term immunotherapy varied widely, reflecting the absences of standardised AE treatment guidelines in Germany. Cortisone pulse therapy (alone or in combination) was initially applied in almost all of our patients which led to improvements in the CASE score.

Relapses were observed more frequently in patients with anti-GAD65 antibodies. Other patients with relapse show extracellular antibodies, which represent the most commonly affected subgroup (8). In patients with intracellular antibodies, interpretation of relapse requires caution due to the typically chronic and fluctuating disease course (27), however we applied a stringent definition of relapse with an increase of ≥ 3 points in the CASE score. In our cohort, approximately one-third of all patients experienced a relapse, consistent with previously reported relapse rates (10- 35%), though it is likely that relapse rates are still underestimated (8).

Notably, patients receiving rituximab as long-term immunotherapy experienced fewer relapses (**Figure 5**). This finding is consistent with previous studies suggesting a reduced relapse risk with rituximab-based long-term immunotherapy (10, 31). Only one patient in the Rituximab group experienced a relapse; this patient had antibodies targeting the GABA(A) receptor. In contrast, the majority of relapses occurred in patients treated with IVIG or those who received no long-term immunotherapy. The limited efficacy of plasmapheresis and IVIG may be attributed to the underlying pathophysiology of AE, as antibody production and inflammatory changes primarily occur behind the blood-brain barrier, where these therapies have limited access (31). Additionally, IVIG was administered to four patients with anti-GAD65 antibodies, a group generally considered to respond less favourably to immunotherapy (32). While these observations suggest a lower relapse rate with rituximab, caution is warranted because the groups included patients with different antibody types and varying relapse risks. The findings are based on a small cohort, primarily consisting of individuals with antibodies against extracellular antigens treated with rituximab, while four of nine patients in the IVIG group had antibodies against GAD65. Consequently, these results are exploratory and should not be interpreted as treatment recommendations, highlighting the need for validation in larger, controlled studies.

Some patients remained relapse free despite receiving alternative immunosuppressive regimens or no long-term immunotherapy at all. Interestingly, patients without long-term immunotherapy showed a better median CASE score during the acute phase of AE. The decision to discontinue long-term immunotherapy may therefore have been influenced by a more favourable initial clinical course.

Overall, determining which patients may benefit from long-term immunosuppressive therapy – and the appropriate duration of such treatment - remains challenging. While some observations in our cohort might suggest that higher antibody titres during the acute phase or persistence of titres in the first few months may be associated with a less favourable course, these findings are exploratory and based on a small number of patients without significant titre differences in the outcome groups. Therefore, no firm conclusions can be drawn regarding the benefit of prolonged immunosuppression, and treatment decisions should continue to be guided primarily by clinical presentation and established guidelines. Further studies could explore whether patients with high titres in the acute phase of AE and persisting antibodies in the first few months might benefit from prolonged therapy, taking into account the different relapse rates depending on the antibody (33). Conversely, in cases with initially low antibody titres and mild clinical symptoms, immunotherapy might be tapered after the acute phase of AE. Emerging therapies, such as bortezomib - which targets CD138-positive long-lived plasma cells - may improve outcomes in patients with persistent antibodies and are currently under investigation in clinical trials (11, 13, 31, 34).

This study has several limitations due to its monocentric and retrospective design. The interpretation of antineuronal antibody titres is limited by the relatively small sample size, and we acknowledge that the study was not designed to comprehensively assess the sensitivity or specificity of antibody testing. Instead, we focussed on patients presenting with neuropsychiatric conditions and positive antibody titres. The evaluation of antibody titres is particularly affected by the small sample size and by confounding factors arising from heterogeneous therapeutic interventions, which may have influenced antibody levels in different ways, given the retrospective nature of the study. The interpretation of treatment effects is limited by substantial heterogeneity in both antibody profiles and therapeutic regimens. In addition to rituximab and IVIG, several patients received other immunosuppressive agents, making it difficult to isolate the effects of individual therapies or to draw firm conclusions regarding the efficacy of specific long-term immunotherapy strategies.

All patient data were obtained from medical records, making the findings dependent on the quality of the documentation and specific clinic. Our AE diagnoses were based on clinical criteria, supported by diagnostic findings including EEG, MRI, and CSF analysis, in addition to antibody testing. Whenever possible, dual testing was performed (see **Table 1**), although this was not feasible for all antibodies, which carries a potential risk of misclassification as AE. There were three exceptions in which antibodies were positive in only one method in our in-house laboratory. In these cases, the diagnosis of AE was assigned based on the combination of clinical criteria, supportive diagnostic findings, and antibody detection, even if dual positivity was not achieved. The retrospective application of the Graus criteria for antibody-positive patients resulted in the exclusion of antibody-negative AE cases. Furthermore, the cohort of non-AE patients with a history of a neuropsychiatric autoimmune-mediated condition was not further characterised beyond demographic data. This group includes patients with polyneuropathy, cerebellar ataxia, epilepsy, delirious syndrome and other conditions. A more detailed characterisation of these patients, for instance using to the classification concept proposed by Abboud et al. (2), could provide additional insights into the role of specific antineuronal antibodies in neurological diseases. Another limitation is the relatively small cohort of patients with AE, which reflects both the low incidence of the disease and the heterogeneity of associated antibodies.

However, the distribution of antibodies in our cohort reflects the real-world clinical spectrum of AE and is thus representative of routine clinical practice, despite the inclusion of heterogenous antibody types. Additionally, the lack of standardised treatment guidelines in Germany has led to a heterogeneous range of therapeutic approaches, complicating the interpretation of treatment outcomes. One of the strengths of this study is that nearly all antibody analyses were conducted in the in-house neurological laboratory, ensuring result comparability. Moreover, the CASE score represents a new tool for the long-term assessment of patients with AE. The study also benefits from a relatively long follow-up period, with the last follow-up occurring approximately three years after disease onset.

This study highlights the urgent need for standardised treatment guidelines to optimise patient care. Future research should include randomised controlled trials with larger patient cohorts to determine the efficacy of specific treatment regimens. In addition, further research is needed to identify biomarkers that influence patient outcomes and to analyse existing evidence, such as the association between specific antibodies, antibody titres and antibody persistence with relapse risk. 

## Data Availability

The original contributions presented in the study are included in the article/**Supplementary Material**. Further inquiries can be directed to the corresponding author.
