# Supplementary material for: Antineuronal antibody titres in autoimmune encephalitis: clinical implications for diagnosis and long-term immunotherapy
Source: Front Immunol. 2026 Mar 10;17:1771609. doi: 10.3389/fimmu.2026.1771609 (PMC13008692; doi:10.3389/fimmu.2026.1771609)
Supplement: Supplementary file 1 [file DataSheet1.pdf]

Supplementary Material

Supplementary Figure 1: Gender distribution in patients with positive antibody testing (AE and non-AE patients)

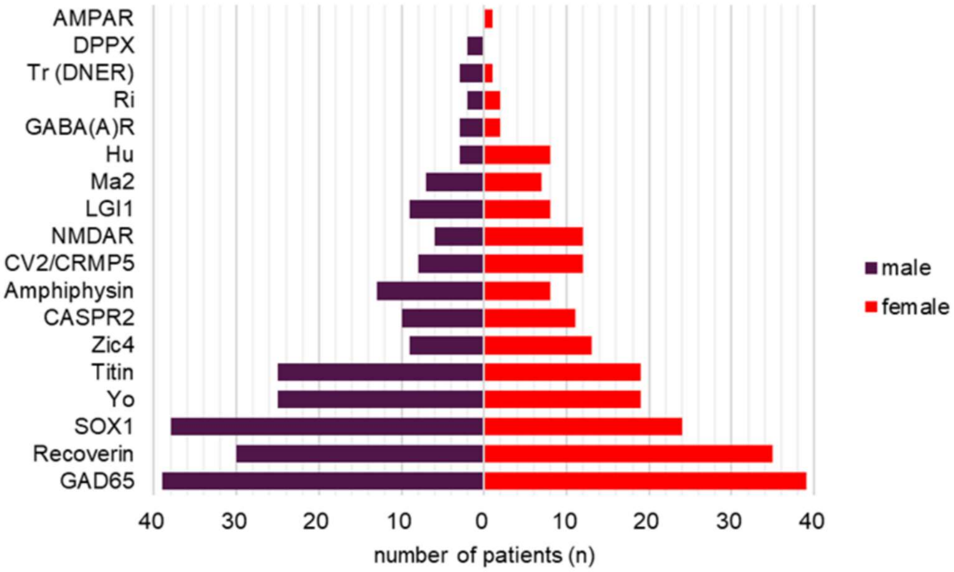

Supplementary Figure 2: Gender distribution in patients with AE; n=53.

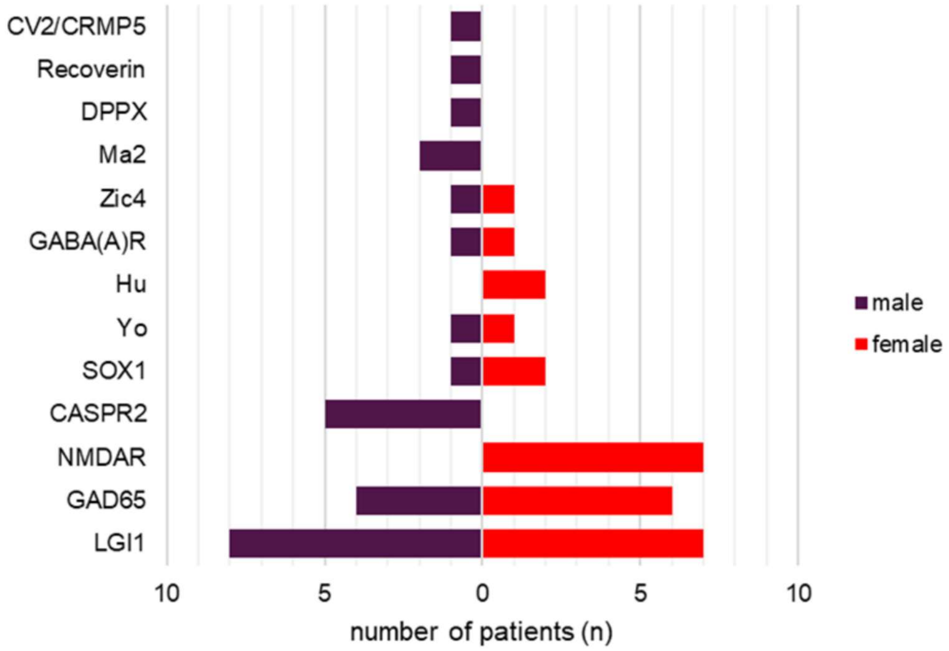

Supplementary Figure 3: Antibody distribution in patients with positive antibody testing (AE and non-AE patients); (n=376).

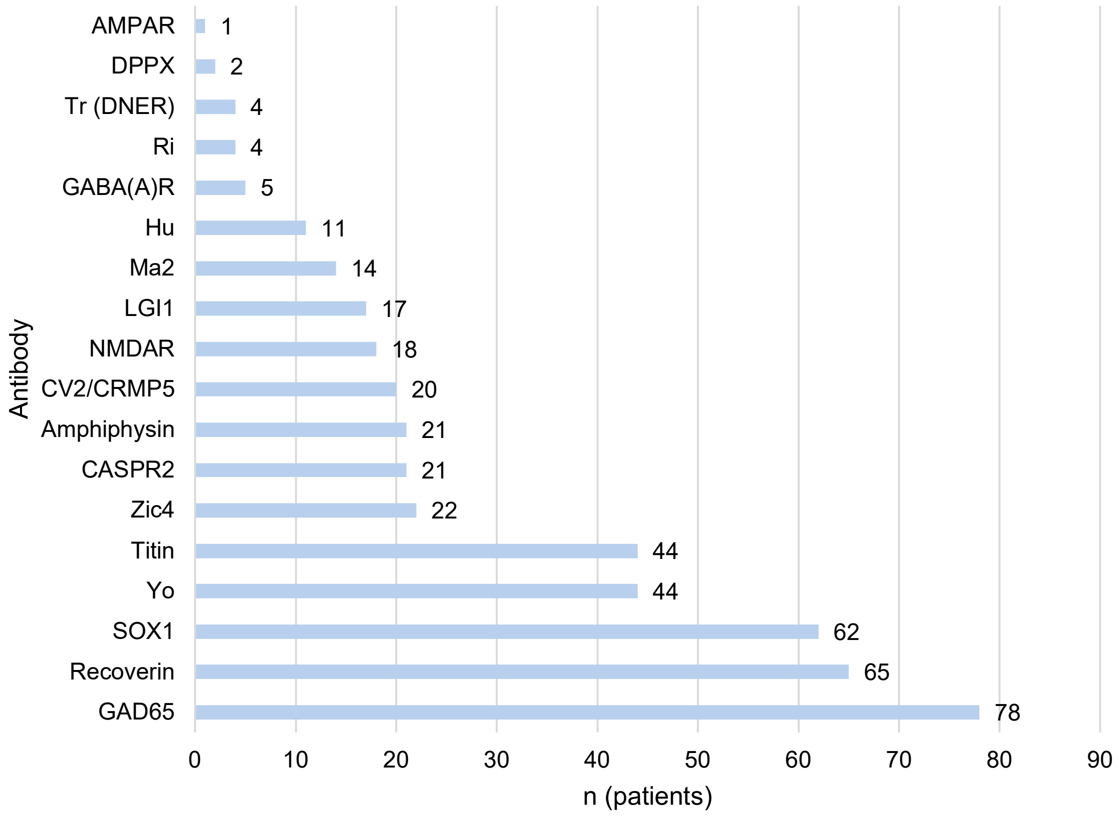

Supplementary Figure 4: Antineuronal antibodies in the AE patient cohort

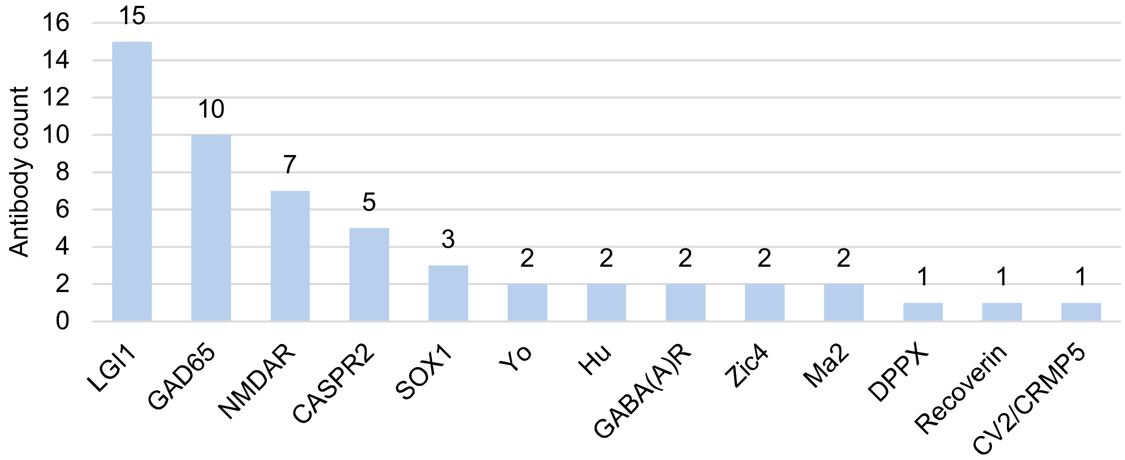

Supplementary Figure 5: Supplementary Figure 1: Correlation of CASE score and mRS at A) acute phase of AE and B) last follow-up.
Overlapping data points are represented as larger points in the diagram.

Abbreviations: MAE= mean absolute error; n= number of patients.

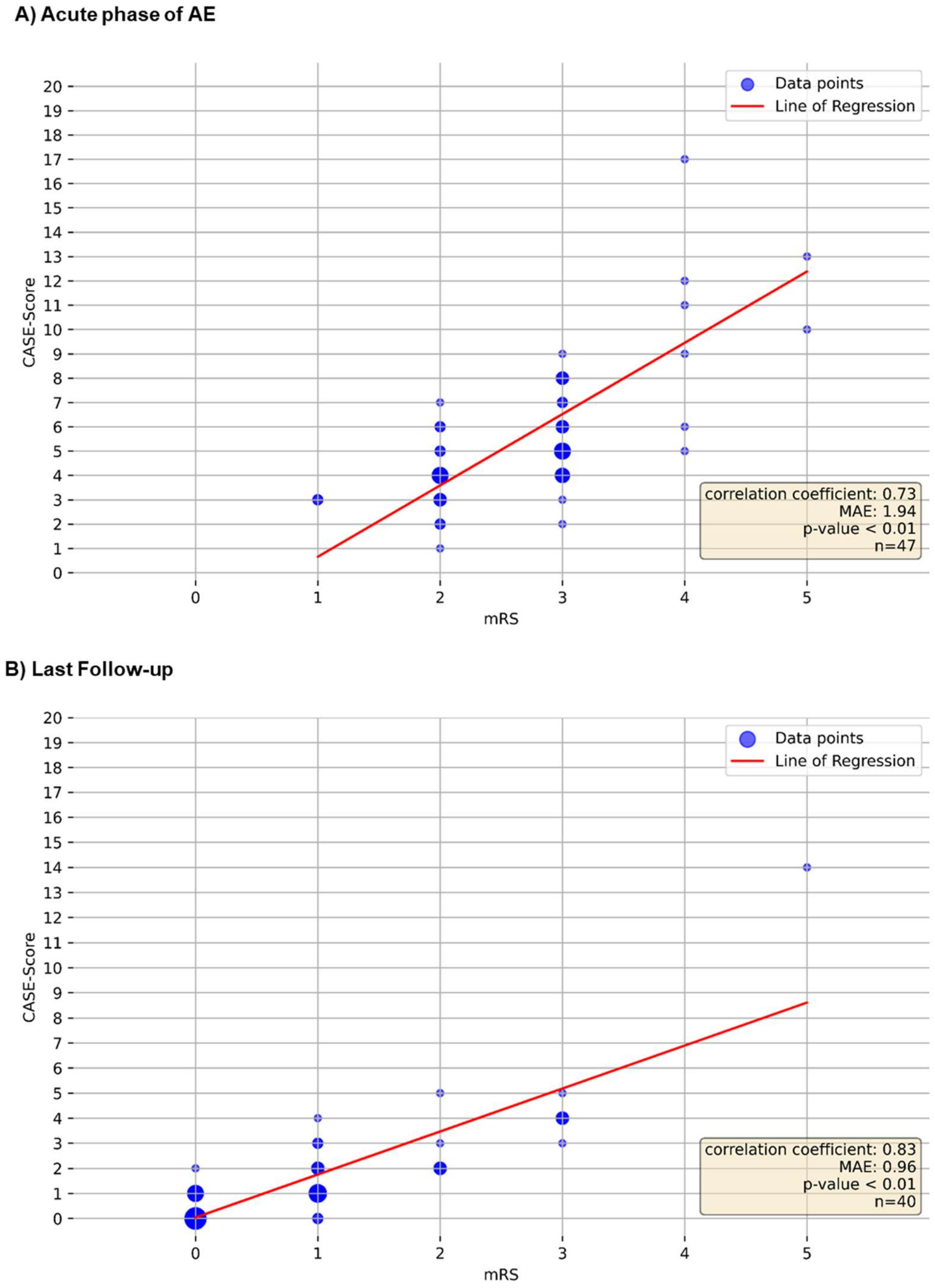

Supplementary Figure 6: Latency of immunotherapy and long-term outcome of patients according to CASE-Score. Ab = Antibody.

Divided into early immunotherapy ('early') <= 4 weeks after symptom onset and late immunotherapy ('late') >= 4 weeks after symptom onset; n (intracellular-AK, patients early) = 3; n (intracellular-AK, patients late) = 5; n (extracellular-AK, patients early) = 9; n (extracellular-AK, patients late) = 17.

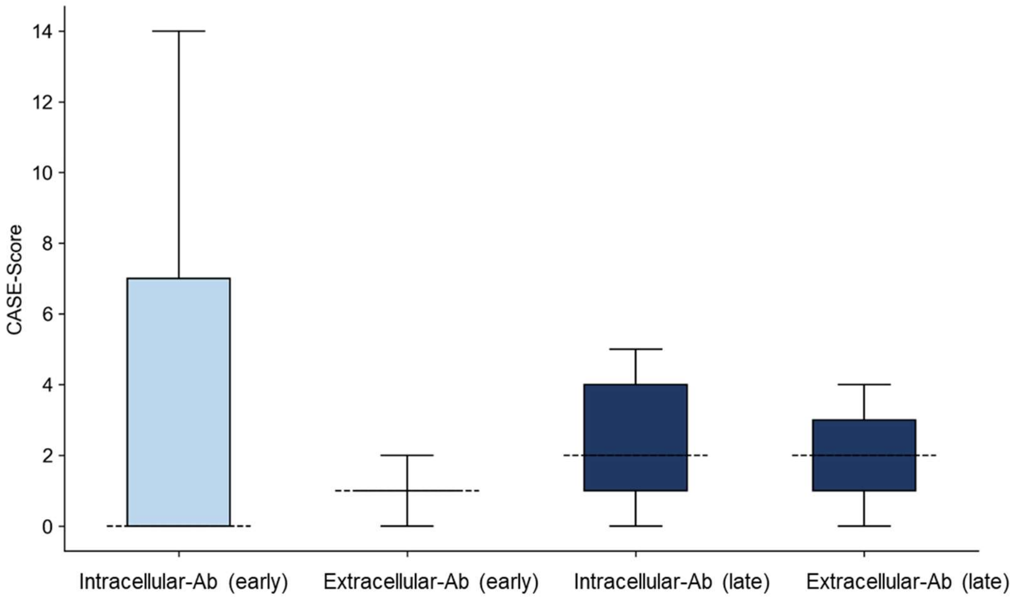

Supplementary Figure 7: Median CASE score over time in patients with intra vs. extracellular antibodies; n = 47; At 0 weeks: n (patients with intracellular antibodies) =18; n (patients with extracellular antibodies) =29; At 188 weeks: n (patients with intracellular antibodies) =4; n (patients with extracellular antibodies) =9.

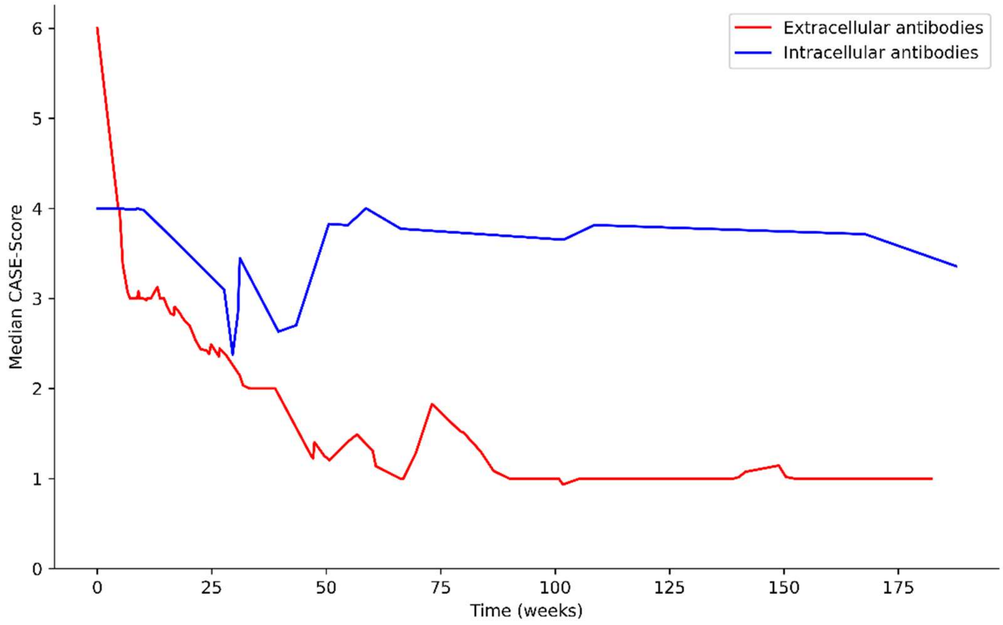

Supplementary Figure 8: Time course of antibody titres in AE patients with relapse in A) CSF and B) serum. Antibody titres are shown on a logarithmic scale (y-axis) at defined time points (x-axis). Each patient is shown with a unique colour–symbol combination; colours indicate antibody targets.

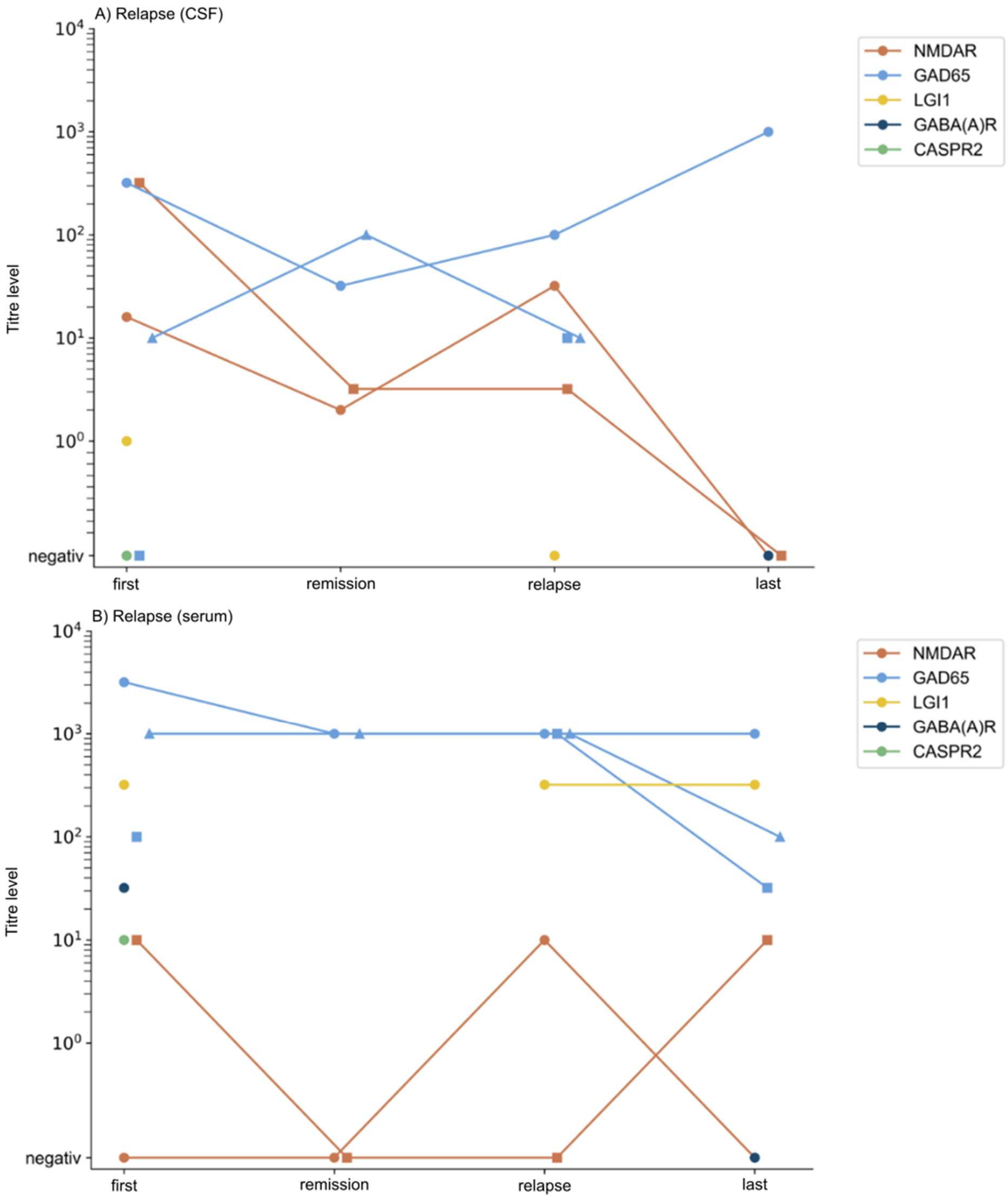

Supplementary Table 1: Fulfilment of Graus criteria for autoimmune encephalitis (n=53).

All patients first met the criteria for possible autoimmune encephalitis, based on a subacute clinical presentation with supportive findings on MRI, EEG and/or CSF after exclusion of alternative causes. In addition, the detection of a disease-specific antineuronal antibody allowed classification as definite autoimmune encephalitis, in accordance with the diagnostic algorithm proposed by Graus et al. (2016).

**Antibody**	**total**	**LGI1**	**GAD65**	**NMDAR**	**CASPR2**	**other**
**Patients (n)**	**53**	**15**	**10**	**7**	**5**	**16**
Subacute onset of working memory deficit (n, %)	40 (75)	13 (87)	8 (80)	5 (71)	3 (60)	11 (69)
Subacute onset of altered mental status (n, %)	28 (53)	6 (40)	5 (50)	7 (100)	3 (60)	7 (44)
Subacute onset of psychiatric symptoms (n, %)	20 (38)	6 (40)	2 (20)	6 (86)	1 (20)	5 (31)
New focal CNS findings (n, %)	50 (94)	13 (87)	10 (100)	7 (100)	5 (100)	15 (94)
Epileptic seizure (not caused by pre-existing epilepsy) (n, %)	31 (58)	11 (73)	6 (60)	4 (57)	2 (40)	8 (50)
CSF pleocytosis (>5/µl) (n, %)	18 (34)	4 (27)	2 (20)	5 (71)	3 (60)	4 (25)
MRI features suggestive of encephalitis (n, %)	20 (38)	10 (67)	1 (10)	2 (29)	2 (40)	5 (31)
Exclusion of alternative causes (n, %)	53 (100)	15 (100)	10 (100)	7 (100)	5 (100)	16 (100)
Detection of an antineuronal antibody (n, %)	53 (100)	15 (100)	10 (100)	7 (100)	5 (100)	16 (100)

Table 2: Patients with multiple antibodies in AE and non-AE patients (n=62).

**Antibody combination**					**n (%)**
GAD65	Recoverin	-	-	-	5 (8)
Amphiphysin	Recoverin	-	-	-	4 (6)

GAD65	CV2	-	-	-	3 (5)
GAD65	SOX1	-	-	-	3 (5)
GAD65	Amphiphysin	-	-	-	2 (3)
GAD65	Titin	-	-	-	2 (3)
GAD65	Tr	-	-	-	2 (3)
GAD65	Yo	-	-	-	2 (3)
Hu	Zic4	-	-	-	2 (3)
SOX1	Recoverin	-	-	-	2 (3)
SOX1	Titin	-	-	-	2 (3)
Yo	Recoverin	-	-	-	2 (3)
Amphiphysin	CV2	-	-	-	1 (2)
Amphiphysin	Yo	-	-	-	1 (2)
CASPR2	CV2	-	-	-	1 (2)
CASPR2	LGI1	-	-	-	1 (2)
CASPR2	Recoverin	-	-	-	1 (2)
CASPR2	Yo	-	-	-	1 (2)
CASPR2	Yo	-	-	-	1 (2)
GAD65	CASPR2	-	-	-	1 (2)
GAD65	NMDAR	-	-	-	1 (2)
Hu	LGI1	-	-	-	1 (2)
Ma2	Titin	-	-	-	1 (2)

NMDAR	Hu	-	-	-	1 (2)
NMDAR	Titin	-	-	-	1 (2)
Ri	GAD65	-	-	-	1 (2)
Ri	Titin	-	-	-	1 (2)
SOX1	Ma2	-	-	-	1 (2)
Titin	Yo	-	-	-	1 (2)
Tr	SOX1	-	-	-	1 (2)
Zic4	Recoverin	-	-	-	1 (2)
Zic4	Titin	-	-	-	1 (2)
Amphiphysin	Recoverin	NMDAR	-	-	1 (2)
LGI1	CV2	Zic4	-	-	1 (2)
Recoverin	SOX1	Tr	-	-	1 (2)
Recoverin	Titin	Yo	-	-	1 (2)
Ri	Recoverin	Titin	-	-	1 (2)
Titin	Zic4	GAD65	-	-	1 (2)
Zic4	CASPR2	SOX1	-	-	1 (2)
Zic4	SOX1	Hu	-	-	1 (2)
Recoverin	CV2	SOX1	Hu	-	1 (2)
Ma2	Recoverin	SOX1	Amphiphysin	-	1 (2)
Hu	CV2	Recoverin	SOX1	Zic4	1 (2)

Supplementary Table 3: Patient age (in years) for each antibody in patients with positive antibody testing (AE and non-AE patients). n (antibodies)= 453.

Antibody	Patient age in years (median, IQR)
Recoverin	65 (47-73)
GAD65	61 (43-70)
SOX1	59 (52-68)
Yo	66 (48-73)
Titin	70 (56-76)
Zic4	59 (53-67)
CV2/CRMP5	68 (51-75)
Amphiphysin	65 (43-74)
CASPR2	62 (53-73)
Ma2	70 (40-75)
LGI1	67 (59-73)
NMDAR	42 (31-67)
Hu	60 (54-74)
GABA(A)R	56 (51-62)
Ri	55 (43-62)
Tr (DNER)	73 (57-80)
DPPX	60 (59-62)
AMPAR	63 (63-63)
total	62 (48-72)

Supplementary Table 4: Antibodies in the non-AE patient cohort

Antibody	Antibody count
GAD65	67
Recoverin	62
SOX1	58
Yo	42
Titin	42
Zic4	20
CASPR2	16
Amphiphysin	19
CV2/CRMP5	18
NMDAR	11
LGI1	3
Ma2	13
Hu	8
GABA(A)R	4
Ri	3
Tr (DNER)	4
DPPX	1
AMPAR	1
total	392

Supplementary Table 5: Positive antibody tests in non-AE patients in IIFA and Line-Blot; n = 392
+ = positive test; s = serum; l = CSF.

	**Only tested in Serum**			**Tested in serum and CSF**			
Antibody	total	IIFT s +	Blot +	total	IIFT s +	IIFT l +	Blot +
Recoverin	20	-	20	42	-	-	42
GAD65	21	3	20	46	10	8	46
SOX1	21	-	21	37	-	-	37
Yo	12	0	12	30	2	1	30
Titin	18	-	18	24	-	-	24
Zic4	6	-	6	14	-	-	14
CV2/CRMP5	5	0	5	13	0	0	13
Amphiphysin	7	0	7	12	0	0	12
CASPR2	3	3	-	13	13	3	-
Ma2	6	0	6	7	0	0	7
LGI1	1	1	-	2	2	0	-
NMDAR	1	1	-	10	9	5	-
Hu	3	0	3	5	1	2	5
GABA(A)R	1	1	-	3	3	0	-
Ri	2	0	2	1	0	0	1
Tr (DNER)	2	0	2	2	0	0	2
DPPX	0	0	-	1	1	0	-
AMPAR	1	1	-	0	0	0	-

total	130	10	122	262	41	19	233

Supplementary Table 6: Clinical features and diagnostic findings of AE patients in acute phase of AE. Abbreviations: y = years; IQR = interquartile range; BBB = Blood Brain Barrier; Q = Quotient; CASE = Clinical Assessment Score for AE; n= number of patients.

**Clinical features at acute phase of AE**	**Total**	**LGI1**	**GAD65**	**NMDAR**	**CASPR2**	**other**
Antibodies (n, %)	53 (100)	15 (28)	10 (19)	7 (13)	5 (9)	16 (25)
Age at diagnosis (y), median (IQR)	59 (45-71)	62 (53-72)	62 (34-72)	31 (20-35)	66 (59-72)	57 (51-68)
Sex (female)	27	7	6	7	0	7
**MRI** (n)	46	13	8	7	5	13
MRI not suggestive for AE (n, %)	24 (52)	2 (15)	7 (88)	5 (71)	3 (60)	7 (54)
FLAIR/T2 hyperintensities of the mesial temporal lobe unilateral (left) (n, %)	3 (7)	1 (8)	0 (0)	0 (0)	1 (20)	1 (8)
FLAIR/T2 hyperintensities of the mesial temporal lobe unilateral (right) (n, %)	2 (4)	2 (15)	0 (0)	0 (0)	0 (0)	0 (0)
FLAIR/T2 hyperintensities in the mesial temporal lobe on both sides (n, %)	11 (24)	5 (38)	1 (13)	2 (29)	0 (0)	3 (23)
FLAIR/T2 hyperintensities of the mesial temporal lobe bilaterally (left > right) (n, %)	2 (4)	0 (0)	0 (0)	0 (0)	1 (20)	1 (8)
FLAIR/T2 hyperintensities of the mesial temporal lobe on both sides (right > left) (n, %)	2 (4)	2 (15)	0 (0)	0 (0)	0 (0)	0 (0)

FLAIR/T2 hyperintensities multifocal/extralimbic (n, %)	6 (13)	2 (15)	0 (0)	0 (0)	1 (20)	3 (23)
**EEG** (n)	40	14	7	6	5	8
Routine EEG (<1h) performed (n, %)	37 (93)	12 (86)	6 (75)	6 (86)	5 (100)	8 (62)
Long-term EEG (>24 hours) performed (n, %)	5 (13)	4 (29)	1 (14)	0 (0)	0 (0)	0 (0)
Normal EEG (n, %)	14 (35)	3 (21)	2 (29)	2 (33)	3 (60)	4 (50)
diffuse slowdown (n, %)	6 (15)	2 (14)	1 (14)	2 (33)	0 (0)	1 (13)
Focal temporal slowing (n, %)	5 (13)	1 (7)	1 (14)	2 (33)	0 (0)	1 (13)
epileptiform discharges (n, %)	16 (40)	6 (43)	5 (71)	2 (33)	0 (0)	3 (38)
Extreme delta brush (n, %)	1 (3)	0 (0)	0 (0)	1 (17)	0 (0)	0 (0)
**CSF** (n)	44	14	7	7	5	11
Cell count (x/µl) (median, IQR)	3 (1-9)	2 (1-4)	4 (2-6)	19 (9-43)	6 (3-20)	3 (1-5)
Protein (mg/dl) (median, IQR)	43 (33-57)	42 (35-57)	43 (32-56)	35 (22-62)	53 (52-56)	43 (39-54)
BBB dysfunction present (n, %)	14 (32)	5 (36)	2 (29)	2 (29)	2 (40)	3 (27)
Q Albumin (1 x 10^-3)) (median, IQR)	6 (5-9)	8 (5-9)	5 (5-7)	5 (4-9)	8 (3-20)	6 (6-9)
Q IgG (1 x 10^-3) (median, IQR)	3 (2-4)	3 (2-5)	2 (2-4)	3 (2-5)	4 (3-6)	3 (2-4)
**CASE score** (n)	47	14	8	7	5	13
CASE score (median, IQR)	5 (4-7)	5 (3-7)	4 (4-6)	7 (5-11)	5 (4-6)	5 (4-6)
**Seizures (n, %)**	28 (60)	11 (79)	5 (63)	3 (43)	2 (40)	7 (54)
None	19	3	3	4	3	6

Controlled seizures	2	-	1	1	-	-
Uncontrolled seizures (requiring additional therapy)	23	11	3	2	1	6
Status epilepticus	3	-	1	-	1	1
**Memory impairment (n, %)**	36 (77)	12 (86)	6 (75)	5 (71)	2 (40)	11 (85)
none	11	2	2	2	3	2
Mild (no impairment of daily activities)	2	1	1	-	-	-
Moderate (impairment of daily activities)	28	8	5	3	2	10
Severe (no memory of the recent past or unable to communicate)	6	3	-	2	-	1
**Psychiatric symptoms (n, %)**	17 (36)	6 (43)	2 (25)	5 (71)	1 (20)	3 (23)
None	30	8	6	2	4	10
Mild (no need for medical intervention, no impairment of daily activities)	8	3	2	-	-	3
Moderate (need for medical intervention, impairment of daily activities)	8	3	-	4	1	-
Severe (constant care due to psychiatric symptoms)	1	-	-	1	-	-
**Impairment of consciousness (n, %)**	11 (23)	1 (7)	1 (13)	4 (57)	2 (40)	3 (23)
Awake (opens eyes spontaneously)	36	13	7	3	3	10
drowsy (opens eyes to voice)	7	1	-	2	1	3

Stupor (opens eyes in pain)	2	-	-	2	-	-
comatose (does not open eyes)	2	-	1	-	1	-
**Speech disorder (n, %)**	27 (57)	6 (43)	6 (75)	5 (71)	3 (60)	7 (54)
None	20	8	2	2	2	6
Mild (slow, but able to formulate sentences)	9	2	2	1	1	3
Moderate (unable to formulate complete sentences)	16	4	3	3	2	4
Severe (unable to communicate)	2	-	1	1	-	-
**Dyskinesia/dystonia (n, %)**	9 (19)	1 (7)	2 (25)	2 (29)	-	4 (31)
None	38	13	6	5	5	9
Mild (does not affect daily activities)	2	-	1	-	-	1
Moderate (interferes with daily activities)	6	1	1	1	-	3
Severe; causes secondary medical problems	1	-	-	1	-	-
**Gait instability and ataxia (n, %)**	20 (43)	5 (36)	3 (38)	3 (43)	3 (60)	6 (46)
Normal	27	9	5	4	2	7
Slight, can walk without assistance	11	4	2	1	1	3
Moderate, assisted walking	6	1	-	1	1	3
Severe, unable to walk	3	-	1	1	1	-
**Brainstem dysfunction (n, %)**	3 (6)	-	1 (13)	1 (14)	-	1 (20)

none	44	14	7	6	5	12
Gaze palsy	-	-	-	-	-	-
Tube feeding	3	-	1	1	-	1
Ventilator due to central hypoventilation	1	-	1	-	-	-
Ventilator due to central hypoventilation	1	-	-	-	-	1
**Paresis (n, %)**	1 (2)	-	-	-	-	1 (8)
none (grade V)	45	14	8	7	5	11
mild (grade IV)	1	-	-	-	-	1
moderate (grade III)	-	-	-	-	-	-
Severe (>= grade II)	-	-	-	-	-	-
**Other symptoms**						
Autonomic dysfunction (n, %)	4 (9)	4 (29)	-	-	-	-
**Cognitive impairment (n, %)**	44 (94)	13 (93)	8 (100)	6 (86)	5 (100)	12 (92)
none	2	1	-	-	-	1
Mild (does not affect daily activities)	4	1	2	-	1	-
Moderate (impairs daily activities)	35	10	5	4	4	12
Severe (daily activities are very limited or impossible)	5	2	1	2	-	-

**Patients with seizure (n)**	28	11	5	3	2	7
Focal aware (n, %)	15 (54)	9 (82)	1 (20)	-	-	5 (71)
Focal impaired awareness (n, %)	14 (50)	5 (45)	2 (40)	1 (33)	2 (100)	4 (57)
Focal to bilateral tonic clonic (n, %)	5 (18)	1 (9)	-	1 (33)	-	3 (43)
Focal non-motor (n, %)	8 (29)	4 (36)	-	-	-	4 (57)
Focal motor (n, %)	10 (36)	4 (36)	1 (20)	-	2 (100)	3 (43)
Generalized tonic-clonic (n, %)	2 (7)	-	-	2 (67)	-	-
Unknown onset motor (n, %)	1 (4)	-	1 (20)	-	-	-
Unkown onset non-motor (n, %)	-	-	-	-	-	-
Facio-brachial dystonic seizure (n, %)	5 (18)	5 (45)	-	-	-	-
Seizures per month (median, IQR)	4 (1-83)	105 (90-600)	1 (1-7)	1 (1-2)	0 (7-18)	2 (2-32)

Supplementary Table 7: Hospitalisation in days and admission to the intensive care unit.

	**total**	**LGI1**	**GAD65**	**NMDAR**	**CASPR2**	**other**
Patients (n)	47	14	8	7	5	13
Time in hospital in days (Median, IQR)	9 (5,75-17,75)	8 (4,5-12,25)	7 (4,75-15)	26 (13,5-29,5)	11 (5-17)	9 (6,5-13,25)
Intensive care unit (n, %)	5 (11)	-	1 (13)	2 (29)	1 (20)	1 (8)

Supplementary Table 8: Pre-existing conditions in AE patients.
Abbreviations: TIA = Transient ischaemic attack; SCLC: small cell lung cancer.

Antibody	Autoimmune	Neurological-Psychiatric	Tumour
LGI1	-	Migraine (n=2)<br>Depression (n=1)<br>Polyneuropathy (n=3)<br>Multiple Sclerosis (n=1)	Adenocarcinoma of the colon (n=1)
GAD65	Diabetes mellitus type 1 (n=2)<br>Thyroid disease (n=2)<br>Psoriasis (n=1)<br>urticaria (n=1)<br>Pernicious anaemia (n=1)	Polyneuropathy (n=1)<br>Hydrocephalus internus, Hippocampus sclerosis (n=1)	Colon carcinoma (n=1)
NMDAR	Autoimmune hepatitis (n=1)	Demyelinating neuropathy (n=1)<br>Depression (n=1)	Teratoma (n=2)
CASPR2		Depression (n=1)<br>TIA (n=1)<br>Migrane (n=1)	Urothelial carcinoma of the urinary bladder (n=1)
SOX1	Thyroid disease (n=1)	-	-
Yo	-	Polyneuropathy (n=1)	Ovarian carcinoma (n=1)
Hu	-	-	SCLC (n=1)

		-	Small cell endocrine cervical carcinoma (n=1)
GABA(A)R	-	-	Small cell neuroendocrine carcinoma of unknown origin (n=1)
Zic4	Systemic vasculitis (n=1)	Media ischaemia, structural epilepsy (n=1)	Neuroendocrine tumour of unclear primary; previously ovarian carcinoma (n=1)
Ma2	-	Polyneuropathy (n=1)	-
DPPX	Psoriasis (n=1)	Depression (n=1)	-
Recoverin	-	TIA (n=1)	-
CV2	-	-	Neuroendocrine tumour of the pancreatic tail (n=1)
**Total diseases**	**8**	**18**	**11**

Supplementary Table 9: Patient age of AE patients by antibody; n (patients) =53.

**Antibody**	**Age in years (Median, IQR)**
LGI1	62 (53-72)
GAD65	62 (34-72)
NMDAR	31 (20-35)
CASPR2	66 (59-72)
more	57 (51-68)
total	59 (45-71)

Supplementary Table 10: Acute therapy in AE patients (n=47)

Response to therapy, defined as clinical improvement at least 4 weeks after treatment initiation, was observed in 86% of patients.

Abbreviations: IVIG= intravenous Immunoglobulins; C= Cortisone; I = IVIG; P = Plasmapheresis; R = Rituximab.

**Antiinflammatory Therapy**	**total**	**LGI1**	**GAD65**	**NMDAR**	**CASPR2**	**other**
**Patients (n)**	**47**	**14**	**8**	**7**	**5**	**8**
no acute therapy received (n, %)	5 (11)	-	-	-	-	5 (63)
1000-2000 mg of intravenous methylprednisolone per day (for three to five days) (n, %)	40 (85)	13 (93)	7 (88)	7 (100)	5 (100)	8 (100)
Oral corticosteroid tapering regimen (n, %)	21 (45)	7 (50)	1 (13)	6 (86)	2 (40)	5 (63)
Intravenous immunoglobulins (IVIG, total dose 2 g/kg body weight), administered as 0.4 g/kg/day over 5 days (n, %)	18 (38)	5 (36)	2 (25)	7 (100)	2 (40)	2 (25)
Plasmapheresis / immunoadsorption (n, %)	7 (15)	1 (7)	1 (13)	2 (29)	-	3 (38)
Rituximab	6 (13)	1 (7)	-	2 (29)	-	3 (38)

Supplementary Table 11: Long-term immunotherapy in AE patients (n = 34) including all therapies used for sustained immunosuppression, before and/or after relapse.
Abbreviations: C = corticosteroid pulse, I = IVIG, R = rituximab, A = azathioprine, M = methotrexate, P = plasmapheresis

	**total**	**LGI1**	**GAD65**	**NMDAR**	**CASPR2**	**other**
**Patients (n)**	34	13	6	6	5	4
no long-term immunotherapy (n, %)	7 (21)	2 (15)	-	1 (17)	2 (40)	2 (50)
**Therapy combinations**						

C (n, %)	2 (6)	1 (8)	1 (17)	-	-	-
I (n, %)	6 (18)	4 (31)	2 (33)	-	-	-
R (n, %)	3 (9)	1 (8)	-	2 (33)	-	-
CI (n, %)	1 (3)	-	-	1 (17)	-	-
CIA (n, %)	1 (3)	-	1 (17)	-	-	-
CPI (n, %)	1 (3)	-	1 (17)	-	-	-
CR (n, %)	2 (6)	-	-	-	1 (20)	1 (25)
CRI (n, %)	1 (3)	1 (8)	-	-	-	-
CRM (n, %)	1 (3)	-	-	-	-	1 (25)
CRP (n, %)	2 (6)	1 (8)	-	1 (17)	-	-
RI (n, %)	2 (6)	1 (8)	-	-	1 (20)	-
RP (n, %)	3 (9)	2 (15)	-	-	1 (20)	-
RPI (n, %)	1 (3)	-	1 (17)	-	-	-
RPIM (n, %)	1 (3)	-	-	1 (17)	-	-

Supplementary Table 12 : Positive antibody tests in AE patients in IIFA and Line-Blot in the acute phase.

n (antibodies) = 45; + = positive test; s = serum; l = CSF; - = not detectable with this method; 0 = negative.

Only patients for whom an antibody titre from our laboratory obtained during the acute phase was available are shown. Some patients were tested externally and had antibody positivity reported in external medical records, but no acute-phase titre from our laboratory was available for inclusion.

	**Only tested in Serum**			**Tested in serum and CSF**			
**Antibody**	**total**	**IIFT s +**	**Blot +**	**total**	**IIFT s +**	**IIFT l +**	**Blot +**

Recoverin				1	-	-	1
GAD65				7	5	4	4
SOX1				3	-	-	3
Yo				2	1	1	2
CV2/CRMP5				1	0	0	1
CASPR2				5	5	3	-
LGI1				14	14	10	-
NMDAR				7	3	7	-
Hu				2	2	2	2
GABA(A)R	1	1	-	1	1	0	-
DPPX				1	1	1	-
**total**	**1**	**1**	**-**	**44**	**32**	**28**	**13**

Supplementary Table 13: Long-term outcome groups according to CASE score at least 24 weeks after the start of treatment; n=34.

good outcome: CASE score <3, poor outcome: CASE score >=3, relapse: increase in CASE score by at least three points between follow-up points.

**Outcome-Group**	**total**	**LGI1**	**GAD65**	**NMDAR**	**CASPR2**	**other**
Patients (n)	34	13	6	6	5	4
Good (n, %)	17 (50)	9 (69)	2 (33)	3 (50)	3 (60)	-
Poor (n, %)	7 (21)	3 (23)	-	-	1 (20)	3 (75)
Relapse (n, %)	10 (29)	1 (8)	4 (67)	3 (50)	1 (20)	1 (25)

Supplementary Table 14: Symptoms according to CASE score and cognitive impairment at last follow-up
Time of last follow-up after start of therapy in years (median; IQR): 2.9; 1.3-4.0.

	total	LGI1	GAD65	NMDAR	CASPR2	other
Patients (n)	34	13	6	6	5	4
Seizure (n, %)	7 (21)	4 (31)	2 (33)	-	-	1 (25)
Memory deficit (n, %)	18 (53)	7 (54)	2 (33)	3 (50)	4 (80)	2 (50)
Psychiatric symptoms (n, %)	4 (12)	-	1 (17)	2 (33)	1 (20)	-
Impairment of consciousness (n, %)	1 (3)	-	1 (17)	-	-	-
Speech disorder (n, %)	5 (15)	1 (8)	1 (17)	-	1 (20)	2 (50)
Dyskinesia/ dystonia (n, %)	2 (6)	-	-	-	-	2 (50)
Gait instability and ataxia (n, %)	10 (29)	3 (23)	2 (33)	-	2 (40)	3 (75)
Brainstem dysfunction (n, %)	-	-	-	-	-	-
Paresis (n, %)	1 (3)	-	-	-	-	1 (25)
Cognitive impairment (n, %)	21 (62)	8 (62)	3 (50)	2 (33)	5 (100)	3 (75)

Supplementary Table 15: Time from onset of symptoms to start of therapy in weeks.

	total	LGI1	GAD65	NMDAR	CASPR2	other
Patients (n)	42	14	8	7	5	8
Median, IQR	5,4 (1,6-22,9)	7,4 (4,6-19,9)	4,7 (1,7-22,5)	0,9 (0,6-2,7)	21,6 (3,1-26,6)	14,5 (4,5-26,5)
